# Perceptions of science, science communication, and climate change attitudes in 68 countries – the TISP dataset

**DOI:** 10.1038/s41597-024-04100-7

**Published:** 2025-01-20

**Authors:** Niels G. Mede, Viktoria Cologna, Sebastian Berger, John Besley, Cameron Brick, Marina Joubert, Edward W. Maibach, Sabina Mihelj, Naomi Oreskes, Mike S. Schäfer, Sander van der Linden, Nor Izzatina Abdul Aziz, Suleiman Abdulsalam, Nurulaini Abu Shamsi, Balazs Aczel, Indro Adinugroho, Eleonora Alabrese, Alaa Aldoh, Mark Alfano, Innocent Mbulli Ali, Mohammed Alsobay, Marlene Altenmüller, R. Michael Alvarez, Richard Amoako, Tabitha Amollo, Patrick Ansah, Denisa Apriliawati, Flavio Azevedo, Ani Bajrami, Ronita Bardhan, Keagile Bati, Eri Bertsou, Cornelia Betsch, Apurav Yash Bhatiya, Rahul Bhui, Olga Białobrzeska, Michał Bilewicz, Ayoub Bouguettaya, Katherine Breeden, Amélie Bret, Ondrej Buchel, Pablo Cabrera-Álvarez, Federica Cagnoli, André Calero Valdez, Timothy Callaghan, Rizza Kaye Cases, Sami Çoksan, Gabriela Czarnek, Steven De Peuter, Ramit Debnath, Sylvain Delouvée, Lucia Di Stefano, Celia Díaz-Catalán, Kimberly C. Doell, Simone Dohle, Karen M. Douglas, Charlotte Dries, Dmitrii Dubrov, Małgorzata Dzimińska, Ullrich K. H. Ecker, Christian T. Elbaek, Mahmoud Elsherif, Benjamin Enke, Tom W. Etienne, Matthew Facciani, Antoinette Fage-Butler, Md. Zaki Faisal, Xiaoli Fan, Christina Farhart, Christoph Feldhaus, Marinus Ferreira, Stefan Feuerriegel, Helen Fischer, Jana Freundt, Malte Friese, Simon Fuglsang, Albina Gallyamova, Patricia Garrido-Vásquez, Mauricio E. Garrido Vásquez, Winfred Gatua, Oliver Genschow, Omid Ghasemi, Theofilos Gkinopoulos, Jamie L. Gloor, Ellen Goddard, Mario Gollwitzer, Claudia González-Brambila, Hazel Gordon, Dmitry Grigoryev, Gina M. Grimshaw, Lars Guenther, Håvard Haarstad, Dana Harari, Lelia N. Hawkins, Przemysław Hensel, Alma Cristal Hernández-Mondragón, Atar Herziger, Guanxiong Huang, Markus Huff, Mairéad Hurley, Nygmet Ibadildin, Maho Ishibashi, Mohammad Tarikul Islam, Younes Jeddi, Tao Jin, Charlotte A. Jones, Sebastian Jungkunz, Dominika Jurgiel, Zhangir Kabdulkair, Jo-Ju Kao, Sarah Kavassalis, John R. Kerr, Mariana Kitsa, Tereza Klabíková Rábová, Olivier Klein, Hoyoun Koh, Aki Koivula, Lilian Kojan, Elizaveta Komyaginskaya, Laura König, Lina Koppel, Kochav Koren Nobre Cavalcante, Alexandra Kosachenko, John Kotcher, Laura S. Kranz, Pradeep Krishnan, Silje Kristiansen, André Krouwel, Toon Kuppens, Eleni A. Kyza, Claus Lamm, Anthony Lantian, Aleksandra Lazić, Oscar Lecuona, Jean-Baptiste Légal, Zoe Leviston, Neil Levy, Amanda M. Lindkvist, Grégoire Lits, Andreas Löschel, Alberto López-Ortega, Carlos Lopez-Villavicencio, Nigel Mantou Lou, Chloe H. Lucas, Kristin Lunz-Trujillo, Mathew D. Marques, Sabrina J. Mayer, Ryan McKay, Hugo Mercier, Julia Metag, Taciano L. Milfont, Joanne M. Miller, Panagiotis Mitkidis, Fredy Monge-Rodríguez, Matt Motta, Iryna Mudra, Zarja Muršič, Jennifer Namutebi, Eryn J. Newman, Jonas P. Nitschke, Ntui-Njock Vincent Ntui, Daniel Nwogwugwu, Thomas Ostermann, Tobias Otterbring, Jaime Palmer-Hague, Myrto Pantazi, Philip Pärnamets, Paolo Parra Saiani, Mariola Paruzel-Czachura, Michal Parzuchowski, Yuri G. Pavlov, Adam R. Pearson, Myron A. Penner, Charlotte R. Pennington, Katerina Petkanopoulou, Marija M. Petrović, Jan Pfänder, Dinara Pisareva, Adam Ploszaj, Karolína Poliaková, Ekaterina Pronizius, Katarzyna Pypno-Blajda, Diwa Malaya A. Quiñones, Pekka Räsänen, Adrian Rauchfleisch, Felix G. Rebitschek, Cintia Refojo Seronero, Gabriel Rêgo, James P. Reynolds, Joseph Roche, Simone Rödder, Jan Philipp Röer, Robert M. Ross, Isabelle Ruin, Osvaldo Santos, Ricardo R. Santos, Philipp Schmid, Stefan Schulreich, Bermond Scoggins, Amena Sharaf, Justin Sheria Nfundiko, Emily Shuckburgh, Johan Six, Nevin Solak, Leonhard Späth, Bram Spruyt, Olivier Standaert, Samantha K. Stanley, Gert Storms, Noel Strahm, Stylianos Syropoulos, Barnabas Szaszi, Ewa Szumowska, Mikihito Tanaka, Claudia Teran-Escobar, Boryana Todorova, Abdoul Kafid Toko, Renata Tokrri, Daniel Toribio-Florez, Manos Tsakiris, Michael Tyrala, Özden Melis Uluğ, Ijeoma Chinwe Uzoma, Jochem van Noord, Christiana Varda, Steven Verheyen, Iris Vilares, Madalina Vlasceanu, Andreas von Bubnoff, Iain Walker, Izabela Warwas, Marcel Weber, Tim Weninger, Mareike Westfal, Florian Wintterlin, Adrian Dominik Wojcik, Ziqian Xia, Jinliang Xie, Ewa Zegler-Poleska, Amber Zenklusen, Rolf A. Zwaan

**Affiliations:** 1 Department of Communication and Media Research, University of Zurich, Zurich, Switzerland; 2 Department of the History of Science, Harvard University, Cambridge, USA; 3 Department of Environmental Systems Science, ETH Zurich, Switzerland; 4 Institute of Sociology, University Bern, Bern, Switzerland; 5 Department of Advertising+Public Relations, Michigan State University, East Lansing, USA; 6 Department of Psychology, University of Amsterdam, Amsterdam, The Netherlands; 7 Department of Psychology, Inland Norway University of Applied Sciences, Lillehammer, Norway; 8 Centre for Research on Evaluation, Science and Technology, Stellenbosch University, Stellenbosch, South Africa; 9 Centre for Climate Change Communication, George Mason University, Fairfax, USA; 10 Department of Communication and Media, Loughborough University, Loughborough, UK; 11 Department of Psychology, University of Cambridge, Cambridge, UK; 12 Institute of Malaysian and International Studies, National University of Malaysia, Bangi, Malaysia; 13 School of Collective Intelligence, Mohammed VI Polytechnic University, Ben Guerir, Morocco; 14 Department of Science and Technology Studies, Faculty of Science, Universiti Malaya, Kuala Lumpur, Malaysia; 15 ELTE Institute of Psychology, Eotvos Lorand University, Budapest, Hungary; 16 School of Psychology, University of Sheffield, Sheffield, UK; 17 Faculty of Psychology, Atma Jaya Catholic University of Indonesia, Jakarta, Indonesia; 18 Department of Economics, University of Bath, Claverton Down, UK; 19 Department of Philosophy, Macquarie University, Macquarie Park, Australia; 20 Department of Biochemistry, Faculty of Science, University of Dschang, Cameroun, Cameroon; 21 Sloan School of Management, Massachusetts Institute of Technology, Cambridge, USA; 22 Department of Psychology, LMU Munich, Munich, Germany; 23 Leibniz Institute for Psychology, Trier, Germany; 24 Linde Center for Science, Society, and Policy, Division of Humanities and Social Science, California Institute of Technology, California Institute of Technology, Pasadena, USA; 25 Department of Communication, George Mason University, Fairfax, USA; 26 Department of Physics, Egerton University, Njoro, Kenya; 27 Department of Psychology, Universitas Islam Negeri Sunan Kalijaga, Yogyakarta, Indonesia; 28 Department of Interdisciplinary Social Science, University of Utrecht, Utrecht, The Netherlands; 29 INCT-SANI, National Institute of Science and Technology on Social and Affective Neuroscience, São Paulo, Brazil; 30 Museum of Natural Sciences “Sabiha Kasimati”, University of Tirana, Tirana, Albania; 31 Department of Architecture, University of Cambridge, Cambridge, Cambridge, UK; 32 Department of Biomedical Sciences, University of Botswana, Gaborone, Botswana; 33 Institute of Political Science, University of St. Gallen, Gallen, Switzerland; 34 Institute for Planetary Health Behaviour, University of Erfurt, Erfurt, Germany; 35 Department of Economics, University of Birmingham, Birmingham, UK; 36 Institute for Data, Systems, and Society, Massachusetts Institute of Technology, Cambridge, USA; 37 Institute of Psychology, SWPS University, Warszawa, Poland; 38 Faculty of Psychology, University of Warsaw, Warsaw, Poland; 39 School of Psychology, University of Birmingham, Birmingham, UK; 40 Computer Science Department, Harvey Mudd College, Claremont, USA; 41 Department of Psychology, Nantes Université, Nantes, France; 42 Institute for Sociology, Slovak Academy of Sciences, Staré Mesto, Slovakia; 43 Spanish Foundation for Science and Technology, Madrid, Spain; 44 Department of International and Political Sciences, University of Genoa, Genoa, Italy; 45 Institute for Multimedia and Interactive Systems, University of Lübeck, Lübeck, Germany; 46 Department of Health Law, Policy, and Management, Boston University School of Public Health, Boston, USA; 47 Department of Sociology, University of the Philippines Diliman, Quezon City, Philippines; 48 Department of Psychology, Erzurum Technical University, Erzurum, Türkiye; 49 Network for Economic and Social Trends, Western University, London, Canada; 50 Institute of Psychology, Jagiellonian University, Kraków, Poland; 51 Department of Psychology, KU Leuven, Leuven, Belgium; 52 Cambridge Zero, University of Cambridge, Cambridge, UK; 53 LP3C (Psychology Laboratory), Université Rennes 2, Rennes, France; 54 TRANSOC, Complutense University of Madrid, Madrid, Spain; 55 Department of Cognition, Emotion, and Methods in Psychology, University of Vienna, Vienna, Austria; 56 Institute of General Practice and Family Medicine, University of Bonn, University Hospital Bonn, Bonn, Germany; 57 School of Psychology, University of Kent, Kent, UK; 58 Harding Center for Risk Literacy, University of Potsdam, Potsdam, Germany; 59 Center for Sociocultural Research, HSE University, Moscow, Russia; 60 Department of Labor and Social Policy, University of Lodz, Lodz, Poland; 61 School of Psychological Science & Public Policy Institute, University of Western Australia, Crawley, Australia; 62 Department of Management, Aarhus University, Aarhus, Denmark; 63 Department of Economics, Harvard University, Cambridge, USA; 64 Department of Political Science & Annenberg School for Communication, University of Pennsylvania, Philadelphia, USA; 65 Department of Computer Science and Engineering, University of Notre Dame, Notre Dame, USA; 66 School of Communication and Culture, Aarhus University, Aarhus, Denmark; 67 a2i Programme of ICT Division and UNDP Bangladesh, Dhaka, Bangladesh; 68 Department of Resource Economics and Environmental Sociology, University of Alberta, Edmonton, Canada; 69 Department of Political Science and International Relations, Carleton College, Northfield, USA; 70 Faculty of Management and Economics, Ruhr-University Bochum, Bochum, Germany; 71 LMU Munich School of Management, LMU Munich, Munich, Germany; 72 Leibniz Institut für Wissensmedien, Tübingen, Germany; 73 School of Social Work, Lucerne University of Applied Sciences and Arts, Luzern, Switzerland; 74 Department of Psychology, Saarland University, Saarbrücken, Germany; 75 Department of Political Science, Aarhus University, Aarhus, Denmark; 76 Department of Psychology, Universidad de Concepción, Concepción, Chile; 77 Faculty of Health Sciences, University of Bristol, Bristol, UK; 78 Institute for Management & Organization, Leuphana University, Lüneburg, Germany; 79 School of Psychology, University of New South Wales, Sydney, Australia; 80 UNSW Institute for Climate Risk & Response, University of New South Wales, Sydney, Australia; 81 Research Institute for Responsible Innovation, School of Management, University of St. Gallen, St. Gallen, Switzerland; 82 Department of Business Administration, Instituto Técnológico Autónomo de México, Ciudad de México, Mexico; 83 School of Psychology, Victoria University of Wellington, Wellington, New Zealand; 84 Department of Media and Communication, LMU Munich, Munich, Germany; 85 Department of Geography, University of Bergen, Bergen, Norway; 86 Centre for Climate and Energy Transformation (CET), University of Bergen, Bergen, Norway; 87 Faculty of Data and Decision Sciences, Technion - Israel Institute of Technology, Haifa, Israel; 88 Hixon Center for Climate and the Environment, Harvey Mudd College, Claremont, USA; 89 Faculty of Management, University of Warsaw, Warsaw, Poland; 90 Centro de Investigación y de Estudios Avanzados del Instituto Politícnico Nacional, Mexico City, Mexico; 91 Department of Media and Communication, City University of Hong Kong, Hong Kong, Hong Kong; 92 Department of Psychology, Eberhard Karls Universität Tübingen, Tübingen, Germany; 93 School of Education, Trinity College Dublin, Dublin, Ireland; 94 Department of Political Science and International Relations, KIMEP University, Almaty, Kazakhstan; 95 Center for Integrated Disaster Information Research, Interfaculty Initiative in Information Studies, University of Tokyo, Tokyo, Japan; 96 Department of Government & Politics, Jahangirnagar University, Savar, Bangladesh; 97 Department of Psychology, University of Minnesota, Minneapolis, USA; 98 School of Geography, Planning, and Spatial Sciences, University of Tasmania, Hobart, Australia; 99 Institute of Political Science, University of Bamberg, Bamberg, Germany; 100 Institute of Political Science and Sociology, University of Bonn, Bonn, Germany; 101 Institute of Psychology, Nicolaus Copernicus University, Toruń, Poland; 102 Graduate Institute of Journalism, National Taiwan University, Taipei City, Taiwan; 103 Department of Public Health, University of Otago, Dunedin, New Zealand; 104 Department of Journalism and Mass Communication, Lviv Polytechnic National University, Lviv, Ukraine; 105 Institute of Communication Studies and Journalism, Charles University, Staré Město, Czech Republic; 106 Center for Social and Cultural Psychology, Université Libre de Bruxelles, Bruxelles, Belgium; 107 Department of Political Science and International Relations, Nazarbayev University, Astana, Kazakhstan; 108 Department of Social Research, University of Turku, Turku, Finland; 109 Faculty of Life Sciences: Food, Nutrition and Health, University of Bayreuth, Bayreuth, Germany; 110 Department of Clinical and Health Psychology, University of Vienna, Vienna, Austria; 111 Department of Management and Engineering, Linköping University, Linköping, Sweden; 112 Faculty of Polish and Classical Philology, University of Adam Mickiewicz, Poznań, Poland; 113 Department of Psychology, Ural Federal University, Sverdlovsk, Russia; 114 Department of Information Science and Media Studies, University of Bergen, Bergen, Norway; 115 Department of Communication Science and Political Science, Vrije Universiteit Amsterdam, Amsterdam, The Netherlands; 116 Faculty of Behavioural and Social Sciences, University of Groningen, Groningen, The Netherlands; 117 Department of Communication and Internet Studies, Cyprus University of Technology, Limassol, Cyprus; 118 Laboratoire Parisien de Psychologie Sociale, Université Paris Nanterre, Nanterre, France; 119 Laboratory for Research of Individual Differences, University of Belgrade, Beograd, Serbia; 120 Department of Psychobiology and Methodology, Faculty of Psychology, Universidad Complutense de Madrid, Madrid, Spain; 121 School of Medicine and Psychology, Australian National University, Canberra, Australia; 122 Uehiro Centre for Practical Ethics, University of Oxford, Oxford, UK; 123 Institut Langage et Communication, University of Louvain, Louvain, Belgium; 124 Departamento de Psicología, Universidad Peruana Cayetano Heredia, San Martín de Porres, Peru; 125 Department of Psychology, University of Victoria, Victoria, Canada; 126 Harvard Kennedy School’s Shorenstein Center, Harvard University, Cambridge, USA; 127 Network Science Institute, Northeastern University, Boston, USA; 128 School of Psychology and Public Health, La Trobe University, Bundoora, Australia; 129 Department of Psychology, Royal Holloway, University of London, London, UK; 130 Institut Jean Nicod, Département d’Études cognitives, ENS, EHESS, PSL University, CNRS, Paris, France; 131 Department of Communication, University of Münster, Münster, Germany; 132 School of Psychological and Social Sciences, University of Waikato, Hamilton, New Zealand; 133 Department of Political Science and International Relations, University of Delaware, Newark, USA; 134 Office for Quality Assurance, Analyses and Reporting, Project EUTOPIA, University of Ljubljana, Ljubljana, Slovenia; 135 Department of Management and Supply Chain Studies, Nkumba University, Entebbe, Uganda; 136 Department of Biochemistry and Molecular Biology, University of Buea, Buea, Cameroon; 137 Communication Arts Programme, Bowen University, Ogun, Nigeria; 138 Department of Psychology and Psychotherapy, Witten/Herdecke University, Witten, Germany; 139 Department of Management, University of Adger, Kristiansand, Norway; 140 Faculty of Humanities and Social Sciences, Trinity Western University, Langley Twp, Canada; 141 Department of Clinical Neuroscience, Karolinska Institutet, Solna, Sweden; 142 Institute of Psychology, University of Silesia in Katowice, Katowice, Poland; 143 Penn Center for Neuroaesthetics, University of Pennsylvania, Philadelphia, USA; 144 Institute of Medical Psychology, University of Tuebingen, Tuebingen, Germany; 145 Department of Psychological Science, Pomona College, Claremont, USA; 146 School of Psychology, Aston University, Birmingham, UK; 147 Department of Psychology, University of Crete, Iraklio, Greece; 148 Science Studies Laboratory, University of Warsaw, Warsaw, Poland; 149 Department of Psychology, University of the Philippines Diliman, Quezon City, Philippines; 150 Max Planck Institute for Human Development, Berlin, Germany; 151 Social and Cognitive Neuroscience Laboratory, Mackenzie Presbyterian University, São Paulo, Brazil; 152 Department of Social Sciences, University of Hamburg, Hamburg, 20144 Hamburg Germany; 153 Institut des Géosciences de l’Environnement, University Grenoble Alpes, CNRS, IRD, Grenoble-INP, Saint-Martin-d’Hères, France; 154 Institute of Environmental Health, Lisbon School of Medicine, University of Lisbon, Lisbon, Portugal; 155 Institute of Communication, NOVA University of Lisbon, Lisbon, Portugal; 156 Department of Implementation Research, Bernhard-Nocht-Institute for Tropical Medicine, Hamburg, Germany; 157 Centre for Language Studies, Radboud University Nijmegen, Nijmegen, The Netherlands; 158 Department of Nutritional Sciences, University of Vienna, Vienna, Austria; 159 Department of Cognitive Psychology, Universität Hamburg, Hamburg, Germany; 160 School of Politics and International Relations, Australian National University, Canberra, Australia; 161 TED University, Ankara, Turkey; 162 Independent Researcher, Cairo, Egypt; 163 Département de Sociologie, Université Officielle de Bukavu, Bukavu, Democratic Republic of the Congo; 164 Faculté des Sciences Sociales, Université Catholique de Bukavu, Bukavu, Democratic Republic of the Congo; 165 Sociology Department, Vrije Universiteit Brussel, Brussel, Belgium; 166 Department of Psychology and Neuroscience, Boston College, Boston, USA; 167 Faculty of Political Science and Economics, Waseda University, Shinjuku City, Japan; 168 Department of Civil Law, University of Tirana, Tirana, Albania; 169 Centre for the Politics of Feelings, University of London, London, UK; 170 Division of Public Policy, The Hong Kong University of Science and Technology, Hong Kong, Hong Kong; 171 School of Psychology, University of Sussex, Falmer, UK; 172 Department of Medical Laboratory Science, College of Medicine, University of Nigeria Nsukka, Nsukka, Nigeria; 173 School of Arts, Media and Communiation, UCLan Cyprus, Pyla, Cyprus; 174 Department of Psychology, Education and Child Studies, Erasmus University Rotterdam, Rotterdam, The Netherlands; 175 Department of Psychology, New York University, New York, USA; 176 Faculty of Technology and Bionics, Rhine-Waal University, Kleve, Germany; 177 Melbourne Centre for Behaviour Change, University of Melbourne, Parkville, Australia; 178 Faculty of Philosophy and Social Science, Nicolaus Copernicus University, Toruń, Poland; 179 School of Economics and Management, Tongji University, Shanghai, China; 180 School of Environment, Tsinghua University, Beijing, China

**Keywords:** Society, Psychology, Sociology, Climate change, Communication

## Abstract

Science is integral to society because it can inform individual, government, corporate, and civil society decision-making on issues such as public health, new technologies or climate change. Yet, public distrust and populist sentiment challenge the relationship between science and society. To help researchers analyse the science-society nexus across different geographical and cultural contexts, we undertook a cross-sectional population survey resulting in a dataset of 71,922 participants in 68 countries. The data were collected between November 2022 and August 2023 as part of the global Many Labs study “Trust in Science and Science-Related Populism” (TISP). The questionnaire contained comprehensive measures for individuals’ trust in scientists, science-related populist attitudes, perceptions of the role of science in society, science media use and communication behaviour, attitudes to climate change and support for environmental policies, personality traits, political and religious views and demographic characteristics. Here, we describe the dataset, survey materials and psychometric properties of key variables. We encourage researchers to use this unique dataset for global comparative analyses on public perceptions of science and its role in society and policy-making.

## Background & Summary

Scientific evidence and expertise are fundamental to society. They can inform policy-making, individual decision-making, and public discourse about fundamental challenges to humanity, such as climate change and pandemic response^1^. Yet to effectively fulfil this role, scientists need both to signal trustworthiness and to be perceived as trustworthy by the public^2^. Otherwise science will lose legitimacy and thus be limited in its capacity to provide the best available knowledge to society^3,4^.

Some scholars and pundits, media reports, and empirical studies have concluded that public trust in science is in decline in many countries. They suggest that the epistemic authority of science has been challenged by: politically motivated resentment^5,6^; concerns about scientists illegitimately intruding in policy-making, public debate, and people’s personal lives^7,8^; populist claims about academic elites disregarding common sense in favour of allegedly useless scientific knowledge^9,10^; increased exposure to science-related disinformation and conspiracy theories on social media^11,12^; and scepticism towards scientific evidence and policy advice on major societal issues like climate change^13–15^. This has sparked concerns about a public “breach of faith with science”^16^, but robust evidence is largely missing^17^.

**Fig. 1:**
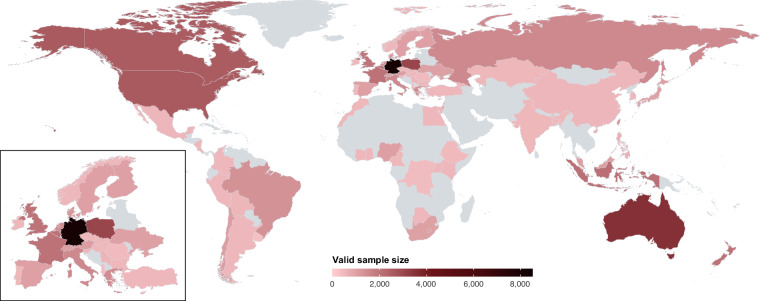
Valid sample size across countries.

**Fig. 2:**
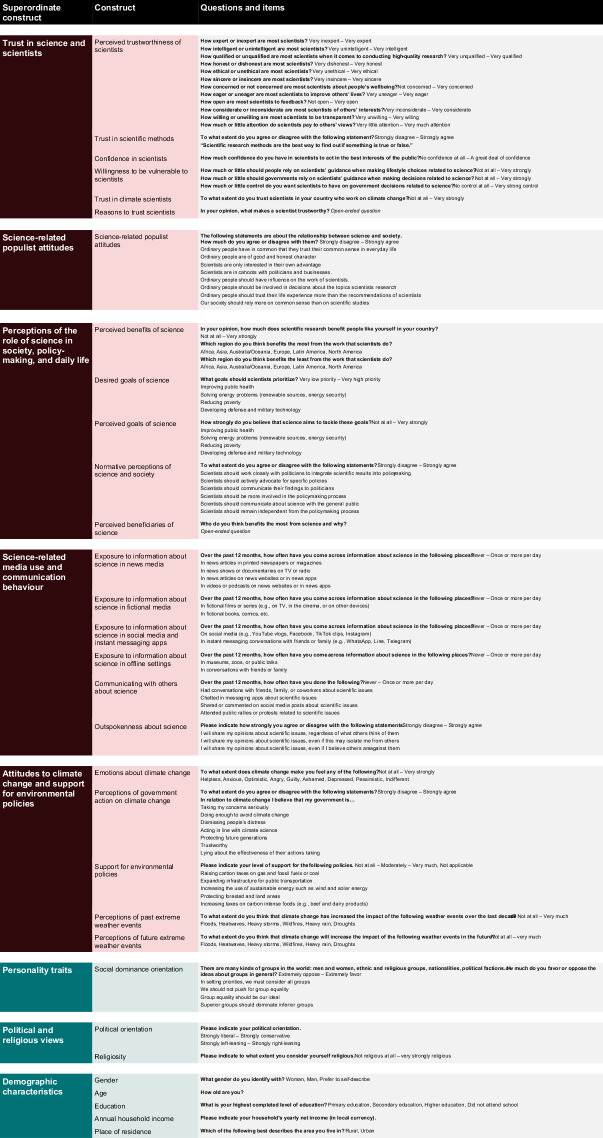
Overview of constructs included in the TISP core questionnaire.

We investigated these concerns with a global, pre-registered, cross-sectional online survey of *N* = 71,922 participants in *k* = 68 countries (see Fig. 1; the term “country” in this article refers to both sovereign states and territories not recognised as such). The survey measured individuals’ (1) trust in science and scientists, (2) science-related populist attitudes, (3) perceptions of the role of science in society, policy-making, and daily life, (4) science-related media use and communication behaviour, (5) attitudes to climate change and support for environmental policies, (6) personality traits, (7) political and religious views and (8) demographic characteristics (see Fig. 2a–d for an overview). In this article, we present the dataset, available in a dedicated repository hosted by the Open Science Framework (OSF) at https://osf.io/5c3qd.

The data were collected between November 2022 and August 2023 as part of the TISP Many Labs project (“Trust in Science and Science-Related Populism”). TISP is an international, multidisciplinary consortium of 241 researchers from more than 170 institutions across all continents. Researchers conducted a pre-tested, pre-registered online survey with 88 post-hoc weighted quota samples in 68 countries, using the same questionnaire translated into 37 languages. The countries cover all inhabited continents, include populations beyond Western, Educated, Industrialised, Rich, and Democratic (WEIRD) societies and represent 31% of all nations worldwide that jointly make up 79% of the global population.

The TISP dataset is a unique resource for global comparative analyses on individual perceptions of science and its role in society and policy-making, science-related media use and communication behaviour, as well as public attitudes to climate change and support for environmental policies. First, the TISP survey provides the first global data on public opinion and communication about science after the COVID-19 pandemic, which had notable and potentially persistent effects on how individuals view science and engage with science-related information^18–20^. Second, it contains well-tested survey scales and comprehensive item inventories for constructs that have previously often only been measured with single items despite their multidimensional structures^21,22^ or have not been measured in global surveys at all, including trust in scientists^23^ and science-related populist attitudes^24^ as well as relevant correlates like outspokenness^25^ and social dominance orientation^26^. Third, the TISP dataset includes data from non-WEIRD countries, which have been underrepresented in social science research despite distinctive local contexts that can affect how people think and communicate about science^27^. The dataset thus offers a valuable opportunity to address an important limitation of extant research, which is that assumptions on public perceptions of science in countries beyond the ‘Global North’ are prone to wrongful generalisations from WEIRD to non-WEIRD countries: For example, studies in WEIRD countries have suggested that trust in science and religiosity are negatively correlated^28^, but investigations of non-WEIRD countries – where Muslim rather than Christian faith may determine religiosity – have shown the opposite^29^. Fourth, the TISP survey accounts for regional and cultural specificities as data collection was mostly led or advised by local collaborators in order to avoid “parachute science” practices^30^.

The TISP dataset allows systematic assessments of public perceptions of science and their predictors and outcomes at a global scale. Cologna *et al*.^31^ as well as an online data visualisation dashboard (https://tisp.shinyapps.io/TISP) present such assessments. Yet, they focus on public trust in science and attitudes towards scientists’ role in society and policy-making – but do not explore numerous further potentials of the TISP dataset, such as analyses of science communication behaviour and climate change attitudes, qualitative analyses with responses to open-ended questions and analyses of single countries.

By publishing the TISP dataset and supplementing materials, we seek to promote its Findability, increase its Accessibility to researchers within and outside academia, enable its Interoperability across different use cases, and foster its Reusability (FAIR)^32^. This will promote an Open Science culture that equally benefits Western and non-Western scholars^33^ and offer a complementary resource for similar datasets presented in this journal^34^ or elsewhere^35^. We also welcome educators to integrate it into under- and postgraduate teaching^36^ and invite researchers across and beyond the social sciences to use it for original and replication studies. These studies will provide further evidence on the relationship of science and society – both across multiple and within single countries. Such evidence can facilitate recommendations for policy-makers, educators, science communication practitioners, and other stakeholders on how to address societal challenges such as science scepticism and climate change.

## Methods

This section explains in detail how the TISP dataset was collected and pre-processed prior to publication. A few of these explanations are also included in other publications of the TISP project^31^ as per the requirements of respective publication outlets. However, the current article presents the most comprehensive description of the methodological procedures underlying the collection of the TISP dataset.

### Ethical compliance

We submitted the study to the Institutional Review Board (IRB) at Harvard University. It received ethical approval from the Area Committee on the Use of Human Subjects at Harvard University in August 2022, which declared it exempt from full IRB review (protocol #IRB22-1046, see https://osf.io/dc5g7). A modified IRB application, which included the full list of countries to be surveyed, was also considered exempt from full IRB review in November 2022 (protocol #IRB22-1046). Moreover, all co-authors made sure the survey was reviewed by their home institution’s IRB in case review was required and approved or declared exempt from full review. They complied with local ethics, norms, and regulations in the countries where the data were collected (see Supplementary Table 1 for an overview). Informed consent was obtained from all participants before taking the survey.

### Pre-registration

We sought to increase the reproducibility and transparency of our study in response to recent calls for a “credibility revolution” within and beyond the social and behavioural sciences^37^. Hence, we followed best Open Science practices and pre-registered at the OSF all methodological procedures underlying the TISP project on 15^th^ November 2022, i.e. prior to collecting data^38^. The pre-registration employed the most comprehensive OSF template developed by Bowman *et al*.^39^ and describes the study design, data collection procedures, variables and sample size, which was rationalised through simulation-based a-priori power analyses^40,41^: https://osf.io/9ksrj. This pre-registration refers to the main TISP publication^31^ while we submitted three further pre-registrations for subsequent publications. The methodological procedures underlying the collection of the TISP dataset can be found in the sections Design Plan, Sampling Plan and Variables.

We deviated from the pre-registered procedures as follows: (1) We exceeded the overall target sample size (*N* = 62,000) as well as the target sample size for some countries (e.g., Germany) thanks to unexpected additional financial resources. We did not reach the target sample size in six countries (Albania, Bangladesh, Bulgaria, Ethiopia, Romania, Uruguay) because local survey panels were too small to recruit enough respondents in all quota groups. (2) The TISP survey covered six countries not mentioned in the pre-registration (Botswana, Cameroon, Côte d’Ivoire, Egypt, Israel, Uganda) as additional collaborators joined the TISP consortium after submitting the pre-registration. Due to unforeseen reasons, such as lack of funding, we could not collect data as planned in five countries (Honduras, Iran, Nepal, Tanzania, Thailand), but exceeded the pre-registered number of countries (*k* = 68). (3) In order to reach our target sample size and accommodate difficulties with obtaining IRB approval, translating and programming the survey or reaching quota goals in single counties, we extended the data collection period beyond the time span indicated in the preregistration, i.e. until August 2023. (4) We had to open quotas in 13 countries with very skewed population distributions for age (e.g., few citizens aged 60 + years) to reach target sample sizes (Albania, Bangladesh, Bolivia, Botswana, Cameroon, Côte d’Ivoire, Ethiopia, Ghana, Indonesia, Kenya, Nicaragua, Uganda, Uruguay). (5) When computing the post-stratification weights via iterative post-stratification (“raking”), we collapsed adjacent age and education strata in single countries. This was because some age and education strata were empty or sparsely populated in several countries, which makes raking impossible or results in extreme weights when applied to data with sparsely populated strata (see *Data pre-processing* section).

### Participants

The TISP dataset contains complete records of *N* = 71,922 participants from 88 samples across *k* = 68 countries. Overall, we collected a total of *N* = 72,135 complete responses but had to delete 213 records from duplicate respondents. Figure 1 and Table 1 show overviews of valid sample sizes in each country.

**Table 1:** Overview of countries, questionnaire languages, polling companies and valid sample sizes across countries.

Country	Language	Polling company	Valid sample size
Albania	Albanian	Bilendi & respondi	377
Argentina	Spanish	Bilendi & respondi	509
Australia	English	Bilendi & respondi	3,560
Austria	German	Bilendi & respondi	1,076
Bangladesh	Bengali	Bilendi & respondi	496
Belgium	French, Flemish	Bilendi & respondi	2,052
Bolivia	Spanish	Bilendi & respondi	548
Botswana	English	Bilendi & respondi	508
Brazil	Portuguese	Offerwise	1,336
Bulgaria	Bulgarian	Bilendi & respondi	497
Cameroon	French, English	MSi	505
Canada	English	Bilendi & respondi	2,535
Chile	Spanish	Bilendi & respondi	1,058
China	Mandarin (simplified)	Bilendi & respondi	526
Colombia	Spanish	Bilendi & respondi	514
Congo DR	French	Bilendi & respondi	408
Costa Rica	Spanish	Bilendi & respondi	573
Côte d’Ivoire	French, English	MSi	514
Cyprus	Greek	Bilendi & respondi	509
Czech Republic	Czech	Bilendi & respondi	502
Denmark	Danish	Bilendi & respondi	1,227
Egypt	Egyptian Arabic	MSi	512
Ethiopia	English	MSi	455
Finland	Finnish	Bilendi & respondi	1,009
France	French	Bilendi & respondi	2,029
Georgia	Georgian	Bilendi & respondi	528
Germany	German	Bilendi & respondi	8,134
Ghana	English	MSi	509
Greece	Greek	Bilendi & respondi	1,449
Hong Kong	Mandarin (traditional)	Bilendi & respondi	599
Hungary	Hungarian	Bilendi & respondi	508
India	English	Bilendi & respondi	502
Indonesia	Indonesian	Bilendi & respondi	2,104
Ireland	English	Bilendi & respondi	506
Israel	Hebrew	Bilendi & respondi	1,049
Italy	Italian	Bilendi & respondi	1,520
Japan	Japanese	Bilendi & respondi	1,004
Kazakhstan	Kazakh	MSi	520
Kenya	English	MSi	513
Malaysia	Malaysian	Bilendi & respondi	1,046
Mexico	Spanish	Bilendi & respondi	532
Morocco	Standard Arabic, Moroccan Arabic	MSi	503
Netherlands	Dutch	Bilendi & respondi	1,427
New Zealand	English	Bilendi & respondi	2,028
Nicaragua	Spanish	Bilendi & respondi	499
Nigeria	English	Bilendi & respondi	1,040
Norway	Norwegian	Bilendi & respondi	513
Peru	Spanish	Bilendi & respondi	513
Philippines	English, Filipino	Bilendi & respondi	661
Poland	Polish	Bilendi & respondi	3,037
Portugal	Portuguese	Bilendi & respondi	502
Romania	Romanian	Kieskompas	444
Russia	Russian	Toloka.Yandex	1,518
Serbia	Serbian	Bilendi & respondi	575
Slovakia	Slovakian	2Muse	543
Slovenia	Slovenian	Bilendi & respondi	528
South Africa	English	Bilendi & respondi	1,027
South Korea	Korean	Bilendi & respondi	500
Spain	Spanish	Bilendi & respondi	1,015
Sweden	Swedish	Bilendi & respondi	1,013
Switzerland	German, Italian, French	Bilendi & respondi	1,018
Taiwan	Mandarin (traditional)	Bilendi & respondi	1,206
Türkiye	Turkish	Bilendi & respondi	508
Uganda	English	MSi	513
Ukraine	Ukrainian	Bilendi & respondi	1,020
United Kingdom	English	Bilendi & respondi; Prolific	2,008
United States of America	English	Bilendi & respondi	2,580
Uruguay	Spanish	Kieskompas	325

The data cover more than a fourth of countries across all inhabited world regions, apart from Sub-Saharan Africa and the Middle East and North Africa, where coverage is lower (21% and 14% respectively). The countries represent 42% of all high-income, 32% of all upper-middle-income, 26% of all lower-middle-income, and 11% of all low-income countries worldwide (according to the World Bank classification^42^).

In most countries, participants were recruited from online panels by the market research company *Bilendi & respondi* and their partners. Working with one market research company allowed us to make sure that the same participants were not sampled twice in countries with multiple samples. Convenience samples were not accepted. In countries not covered by *Bilendi & respondi*, we worked with other data providers (see Table 1).

Participants received vouchers or credit points for finishing the full survey, which they could then redeem or transfer into money. To complete the survey, they had to (1) be at least 18 years old, (2) agree with the terms and conditions of the consent form, (3) belong to a stratum whose quota target had not been met, (4) pass a first attention check of writing “213” into a text box, and (5) pass a second attention check of selecting “strongly disagree” for an extra item in a scale of science-related populist attitudes^43^.

### Procedure

The surveys used crossed quotas for age × gender with balanced target distributions. The age quota had five bins: 20% 18-29 years, 20% 30-39 years, 20% 40-49 years, 20% 50-59 years, 20% 60 years and older. The gender quota had two bins: 50% male, 50% female. It did not include other genders since available population data indicate substantial country differences in how many people identify with, and are willing to disclose, genders other than male or female. Hence, participants who “prefer to self-describe” or “prefer not to say” their gender were not subject to quota requirements (see *Measures* subsection).

The surveys were programmed with the survey software Qualtrics. The .qsf file of the core survey is available at https://osf.io/qd6f3. All data were collected in online surveys, with the exception of the Democratic Republic of the Congo, where trained interviewers conducted face-to-face interviews and recorded responses in Qualtrics, as this was the only data collection solution available from *Bilendi & respondi*.

The project leads prepared several template files, guides and tutorials, including the TISP guidebook; manuals for data collection and the submission of country datasets to a secure, non-commercial cloud storage service; a survey template file (.qsf format) to be imported into Qualtrics; and materials for IRB applications. Moreover, the project leads assisted some collaborators in programming the survey with Qualtrics by hosting video-call workshops. These measures increased the quality, validity and comparability across countries.

**Fig. 3:**
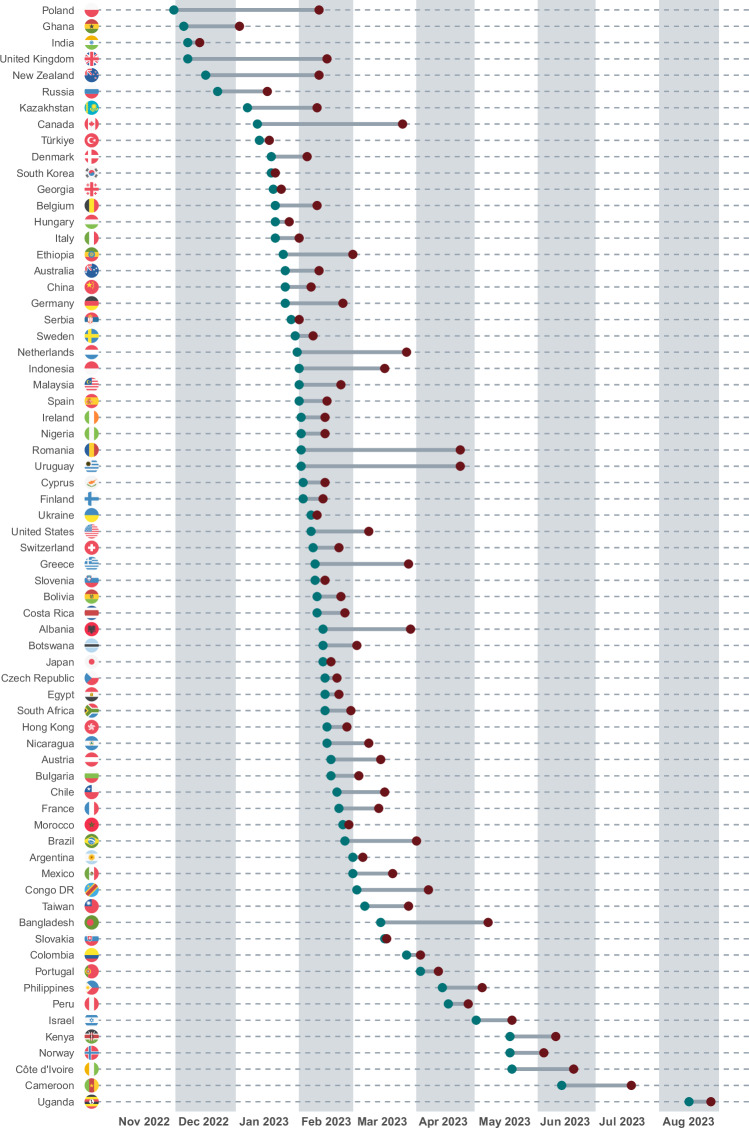
Data collection periods across countries.

Data were collected between 30^th^ November 2022 and 27^th^ August 2023 (see Fig. 3 for an overview of survey periods across countries). The median completion time was 18 minutes (10% winsorised *M* = 21 min, 10% winsorised *SD* = 11 min, *MAD* = 10 min, interquartile range = 14 min).

### Measures

The questionnaire contained 111 variables (see Fig. 2). Data from a few countries missed some variables and items due to negligence or oversight on the part of local collaborators (see Supplementary Table 2 for an overview). However, this pertains only to a small number of variables in eight countries and therefore only marginally impacts the TISP dataset.

The complete questionnaires in all 37 languages and the English core questionnaire are available at OSF: https://osf.io/sujpn. We recommend that users of the TISP dataset refer to the core questionnaire for the labels and codes of variables, items and response options, because a few local questionnaires contained errors. For example, some collaborators used wrong variable and item labels in the local datasets or assigned wrong codes to the response options. However, these errors only concerned the programming back-end of the survey and did not affect questionnaire texts; hence they did not compromise participants’ understanding of the questions. The errors were corrected when preparing the final dataset, but remain in the Qualtrics exports of the original local questionnaires.

The core questionnaire contained the components described in the following (see Fig. 2 for all questions and response options). Participants were presented with these components in the order in which they are explained below, but the order of questions and items of multi-item scales was randomised. Collaborators were allowed to add further measures at the end of the questionnaire in countries where they collected data. Response data for these additional measures are not included in the dataset presented in this paper.

#### Informed consent

Participants were asked to carefully read a consent form (approved under IRB protocol #IRB22-1046 at Harvard University), which included general information about the study and the anonymity of the data.

#### Demographic data I

Participants who agreed to participate in the study indicated their gender (*female*, *male*, *prefer to self-describe*, *prefer not to say*), age (*years*) and education (*did not attend school*, *primary education*, *secondary education, tertiary education*).

#### Attention check I

Participants were asked to write the number “213” into a comment box. Those who failed the attention check were directed to the end of the survey. See the *Technical Validation* section for exclusion totals by country and overall.

#### Definition of science and scientists

Participants were presented with a definition of science and scientists: *When we say “science”, we mean the understanding we have about the world from observation and testing. When we say “scientists”, we mean people who study nature, medicine, physics, economics, history, and psychology, among other things*. This definition was based on the Wellcome Global Monitor^35^. We added it because in-depth interviews conducted by the Monitor suggested that including a definition improves the reliability of cross-country comparisons.

#### Exposure to information about science in news media

Participants were asked how often (*never* – *once or more per day*) they had come across information about science in four types of news media in the past twelve months: news articles in printed newspapers or magazines; news shows or documentaries on TV or radio; news articles on news websites or in news apps; videos or podcasts on news websites or in news apps.

#### Exposure to information about science in fictional media

Participants were asked how often (*never* – *once or more per day*) they had come across information about science in fictional films or TV series and in fictional books, comics, etc. in the past twelve months.

#### Exposure to information about science in social media and instant messaging apps

Participants were asked how often (*never* – *once or more per day*) they had come across information about science on social media and in instant messaging conversations with friends or family in the past twelve months.

#### Exposure to information about science in offline settings

Participants were asked how often (*never* – *once or more per day*) they had come across information about science in museums, zoos or public talks and in conversations with friends or family outside the Internet and messaging apps in the past twelve months.

#### Communicating with others about science

Participants were asked how often (*never* – *once or more per day*) they had communicated about science in four different ways in the past twelve months: having conversations with friends, family, or co-workers about scientific issues; chatting in messaging apps about scientific issues; sharing or commenting on social media posts about scientific issues; attending public rallies or protests related to scientific issues.

#### Open-ended questions on beneficiaries of science and reasons to trust scientists

Participants were randomly assigned to one of two open-ended questions. One question asked participants who they think benefits the most from science and why. The second question asked about their opinion on what makes a scientist trustworthy.

#### Perceived benefits of science

Participants were asked how much they believe that scientific research benefits people like themselves in their country (*not at all* – *very strongly*) and which world region benefits the most and the least from the work that scientists do (*Africa*, *Asia*, *Australia and Oceania*, *Europe*, *Latin America*, *North America*).

#### Desired and perceived goals of science

Participants were asked how much scientists should prioritise tackling four goals (*very low priority* – *very high priority*) and how strongly they believe that science aims to tackle these goals (*not at all* – *very strongly*): improve public health; solve energy problems; reduce poverty; develop defence and military technology.

#### Normative perceptions of science and society

Participants indicated their agreement (*strongly disagree* – *strongly agree*) with six statements about expectations towards the role of science in politics and society, e.g. “Scientists should be more involved in the policy-making process”. Five of these statements were adopted from Cologna *et al*.^44^.

#### Willingness to be vulnerable to scientists

We used three items to measure participants’ willingness to be vulnerable to scientific guidance (*not at all* – *very strongly*), e.g. when making lifestyle choices related to science. Willingness to be vulnerable to others has been conceptualised as a measure of behavioural trust because it reflects the ceding of authority^23^.

#### Perceived trustworthiness of scientists

Trustworthiness of scientists was assessed with twelve questions that are based on Besley *et al*.^23^ and cover four essential dimensions of trust in scientists: competence, integrity, benevolence and openness. The questions used semantic differentials ranging from *very inexpert* (*very dishonest*, *not concerned about people’s well-being*, *not open to feedback* etc.) to *very expert* (*very honest*, *very concerned about people’s well-being*, *very open to feedback* etc.; see Fig. 2). Information on the psychometric properties of the trustworthiness scale, such as its internal consistency, dimensional structure, measurement invariance and convergent validity, can be found in the *Technical Validation* section.

We preferred a multidimensional measure of trust in scientists over unidimensional or single-item measures to capture the multiple conceptual components of trust in science^22^. We opted for the four-dimensional approach of Besley *et al*.^23^ instead of three-dimensional trustworthiness measures like the Muenster Epistemic Trustworthiness Inventory (METI)^45^, because it lacks the openness dimension. Being perceived as open to feedback, willing to be transparent, and considerate of other views are important for scientists in modern societies, where scholars are increasingly expected to be receptive to public demands and engage in dialogical science communication^21^.

#### Trust in scientific methods

Participants indicated how much they agreed that scientific research methods are the best way to find out if something is true or false (*strongly disagree* – *strongly agree*)^46^.

#### Confidence in scientists

Participants were asked how much confidence they have that scientists act in the best interests of the public (*no confidence at all* – *a great deal of confidence*)^47^.

#### Outspokenness about science

We used three items to measure how outspoken participants are about scientific issues, e.g., “I will share my opinions about scientific issues, regardless of what others think of them” (*strongly disagree* – *strongly agree*). These were based on McKeever *et al*.^25^ but reworded so that they referred to scientific issues.

#### Science-related populist attitudes

Science-related populist attitudes were assessed with the SciPop Scale^24^, which measures to what extent individuals believe that scientists represent a corrupt academic elite that allegedly ignores the common sense of ‘ordinary people’^9^. The SciPop Scale asks for the level of agreement with eight statements that capture the four conceptual dimensions of science-related populist attitudes, i.e. positive conceptions of an ordinary people (“Ordinary people have in common that they trust their common sense in everyday life” and “Ordinary people are of good and honest character”), negative conceptions of an academic elite (“Scientists are only interested in their own advantage” and “Scientists are in cahoots with politicians and businesses”), demands for decision-making sovereignty (“Ordinary people should have influence on the work of scientists” and “Ordinary people should be involved in decisions about the topics scientists research”) and demands for truth-speaking sovereignty (“Ordinary people should trust their life experience more than the recommendations of scientists” and “Our society should rely more on common sense than on scientific studies”) on 5-point Likert scales *(strongly disagree* – *strongly agree*). Information on the psychometric properties and measurement performance of the SciPop Scale in the TISP data can be found in the *Technical Validation* section.

#### Attention check II

We integrated a second attention check into the SciPop Scale. It asked participants to select the response option “strongly disagree”. Participants who did not select “strongly disagree” were directed to the end of the survey. See *Technical Validation* section for exclusion totals.

#### Social dominance orientation

To assess social dominance orientation (SDO), we asked participants how much they oppose or favour four statements adopted from Pratto *et al*.^26^, e.g. “In setting priorities, we must consider all groups” (*extremely opposed* – *extremely favour*).

#### Trust in climate scientists

Participants were asked how much they trust scientists in their country who work on climate change (*not at all* – *very strongly*).

#### Emotions about climate change

Participants reported to what extent climate change makes them feel nine emotions: helpless; anxious; optimistic; angry; guilty; ashamed; depressed; pessimistic; indifferent (*not at all* – *very strongly*). Most of the nine items were based on established measures for climate change emotions, such as those developed by Hogg *et al*.^48^ and Searle and Gow^49^.

#### Perceptions of government action on climate change

Following Hickman *et al*.^50^, participants indicated their level of agreement with seven statements about government action on climate change, e.g. “My government is doing enough to avoid climate change” (*strongly disagree* – *strongly agree*).

#### Support for environmental policies

Participants indicated how much they support five environmental policies: raise carbon taxes on gas and fossil fuels or coal; expand infrastructure for public transportation; increase the use of sustainable energy such as wind and solar energy; protect forested and land areas; increase taxes on carbon intense foods (*not at all* – *very much*, *not applicable*).

#### Perceptions of extreme weather events

Participants indicated to what extent they believe that climate change has increased the impact of six weather events over the last decades: floods; heatwaves; heavy storms; wildfires; heavy rain; droughts (*not at all* – *very much*). They also indicated to what extent they expect that climate change will increase the impact of these events in the future (*not at all* – *very much*).

#### Demographic data II and political and religious views

Participants indicated their household’s annual net income (in local currency), their political orientation on the liberal-conservative spectrum (*strongly liberal* – *strongly conservative*, *I don’t know*) and on the left-right spectrum (*strongly left-leaning* – *strongly right-leaning, I don’t know*), as well as their religiosity (*not religious at all* – *very strongly religious*), and whether they live in a rural or urban area (*rural*, *urban*).

### Translations

The questionnaire was prepared in 37 languages. The core questionnaire was developed in English and was used in countries where English is a widely spoken language. In other countries, the questionnaire was translated into local languages and dialects: Albanian, Egyptian Arabic, Modern Arabic, Standard Arabic, Bengali, Bulgarian, Czech, Danish, Dutch, Filipino, Finnish, French, Georgian, German, Greek, Hebrew, Hungarian, Indonesian, Italian, Japanese, Kazakh, Korean, Mandarin (simplified), Mandarin (traditional), Norwegian, Polish, Portuguese, Romanian, Russian, Serbian, Slovak, Slovenian, Spanish, Swedish, Turkish and Ukrainian (see Table 1 for an overview). The survey was usually conducted in a widely spoken language, and in some multilingual countries such as Switzerland, respondents could choose between different national languages.

Most translations were done by researchers from the countries where the surveys were conducted. This allowed us to account for local specificities, such as the Japanese custom to indicate income in “man-Yen”, i.e. in ten thousands of Yen. Collaborators were instructed to ask for permission from the project leads before making any adjustments that could potentially affect comparability across countries. More substantial changes – in particular, the use of gender-neutral language instead of masculine (pro)nouns in countries like Germany – also had to be approved by the project leads.

To maintain the accuracy and consistency of translations, many TISP collaborators cross-checked translations among each other, carried out back-translations, consulted external experts, used validated existing translations when available (e.g., of the SciPop Scale^24^) and worked together to coordinate translations of questionnaires that were used in multiple countries (e.g., the German translation was used in Germany, Switzerland and Austria). Collaborators were advised to apply the highest standards when preparing the translations, such as back-translations by independent researchers. However, the project leads did not require them to employ external back-translations in order to facilitate the project progress and accommodate limited budgets.

These measures enabled us to achieve as much semantic invariance as possible across different translations. However, there are still cross-cultural differences in the meaning of key terms like “science”. For example, the Polish translation “nauka” also means learning, the German translation “Wissenschaft” also includes the humanities, and the Japanese translation “科学” may also be associated with technology and engineering. To mitigate these differences, we placed a definition of the terms “science” and “scientists” at the beginning of the questionnaire. It paraphrased the English meaning of the term, which includes the natural sciences but excludes the arts and humanities (see *Measures* section). We also gave participants examples for “scientific issues” (climate change, vaccination, nutrition, new technologies) and “public rallies or protests related to scientific issues” (COVID-19 protests, Fridays for Future demonstrations, March for Science) to facilitate a common understanding of these terms.

### Data pre-processing

**Fig. 4:**
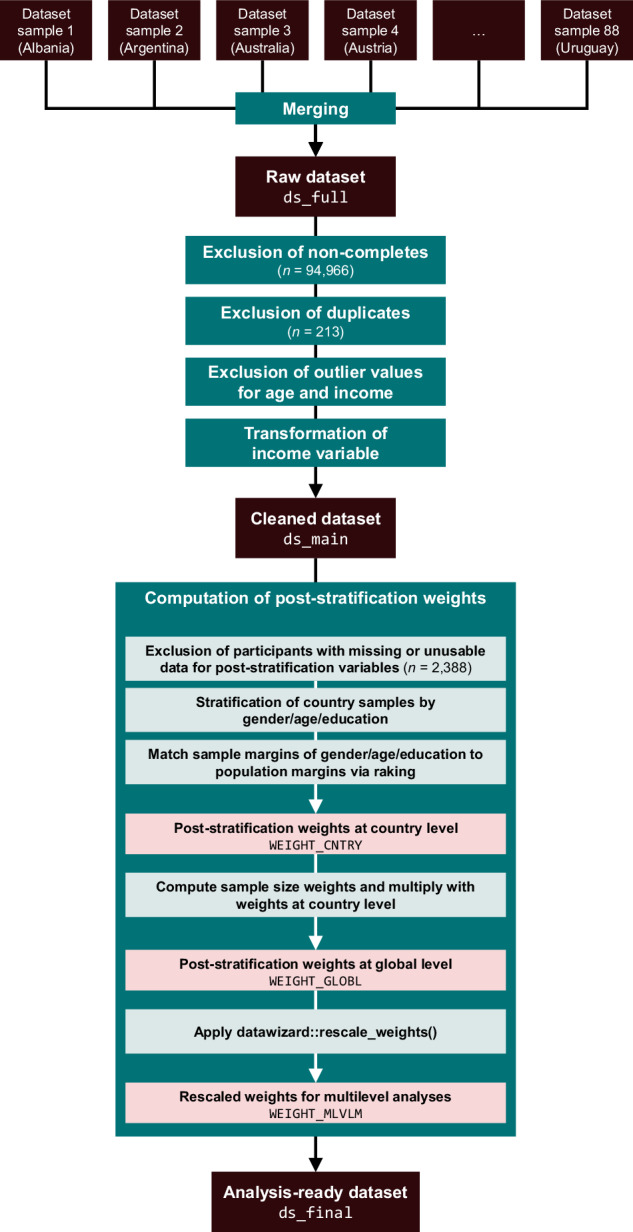
Flow chart of data pre-processing steps.

This section describes how we pre-processed the TISP data to obtain a cleaned dataset without weights (file ds_main in the 01_data/survey-data folder of the OSF repository) and the analysis-ready dataset including post-stratification weights (ds_final in the same folder)^51^. We share both these datasets as well as the raw data (ds_full in the same folder), as explained the *Data Records* section. Figure 4 presents a flow chart visualising the pre-processing steps.

#### Merging and exclusion of non-completes

All research groups of the TISP consortium submitted the collected data to the project leads, including data from participants who did not finish the survey. The final TISP dataset was prepared in the following steps. First, we merged all 88 local datasets into a single dataset (ds_full, see *Data Records* section). We then excluded the 94,966 respondents who did not complete the survey because they cancelled participation during the survey, were filtered out as their gender or age quota were already met or because they did not pass one of the two attention checks.

#### Exclusion of duplicate respondents

Second, we excluded 213 participants who completed the survey more than once despite countermeasures (e.g., IP address checks). We identified these participants by their panel IDs, which they had been assigned by the survey companies when entering the survey, retained only the first complete record for each duplicate respondent and deleted all subsequent records.

#### Outlier exclusion

Third, we removed extreme outlier values for age and household income. Age outliers were defined as values less than 18 and more than 100. Income outliers were defined as values that were smaller than zero (implausible), equal to zero (forbids logarithmic transformation as log 0 is undefined, see *Variable transformations* section) or outside 5 × the interquartile range of the log-transformed income distribution within each country after exclusion of values smaller than zero or equal to zero (which is much more conservative than established outlier definitions^52^ and affects, for example, only highly implausible values of well over 1 billion USD in some countries). This led to the removal of the age values of 8 participants and the removal of the income values of 2,457 participants (1,365 participants indicated income values equal to or less than 0; and 1,092 participants indicated income values outside 5 × the interquartile range). Users who prefer other outlier exclusion criteria or no exclusion at all can adjust the R code to their preferences (file 01_setup.R) and run it on the raw dataset (ds_full).

#### Variable transformations

Fourth, we transformed participants’ annual household income. We converted all values from local currencies to U.S. dollars, using the exchange rates of the day the data were collected. Because almost all countries’ data followed a Pareto distribution, we log-transformed the converted income values, which is beneficial to the robustness of linear regressions that users of the TISP dataset might want to apply^53^. Both the original and transformed income data are contained in the pre-processed datasets (ds_main and ds_final, see *Data Records* section)^51^.

#### Post-stratification weights

Fifth, we used the R package *survey* (v4.4-2)^54^ to compute post-stratification weights for the analysis-ready dataset (ds_final). These ensure that statistical analyses with the TISP data will estimate parameters that are representative for target populations in terms of gender, age and education and have precise standard errors (SEs). We used iterative post-stratification^55^ known as “raking” to compute three kinds of weights, i.e. (1) post-stratification weights at country level, (2) post-stratification weights at global level and (3) rescaled post-stratification weights for multilevel analyses (see *Data Records* section for information on when to use which weight).

We first stratified each country sample by gender (female/male), age groups (18–29/30–39/40–49/50–59/60+ years) and education levels (none or primary education/secondary education/tertiary education). We originally planned to distinguish a *no education* and a *primary education* stratum. However, we had to collapse these into a *none or primary education* stratum, because there were several countries without respondents with no education, making post-stratification impossible. This was a necessary deviation from the preregistration.

We then used raking to match gender, age and education distributions of all country samples to each country’s population margins. Population margins for gender and age were retrieved from the World Population Prospects 2022 of the United Nations^56^ (https://population.un.org/wpp/Download/Files/5_Archive/WPP2022-Excel-files.zip). Population margins for education were retrieved from the 2021 Barro-Lee dataset^57,58^, which contains data on educational attainment for all countries included in the TISP project except Georgia, Ethiopia and Nigeria (https://barrolee.github.io/BarroLeeDataSet/BLData/BL_v3_MF1564.xls). For Georgia, we used 2019 data from the database of the United Nations Economic Commission for Europe^59^ (https://w3.unece.org/PXWeb2015/sq/3290abae-0120-418f-a681-132d4da8f088). For Ethiopia and Nigeria, we used 2011 and 2006 data from the UNESCO Institute for Statistics^60^ (https://uis.unesco.org/sites/default/files/documents/bdds/022024/SDG.zip).

Some age and education strata were empty or sparsely populated in several countries, because collaborators had to relax age quotas or oversampled individuals with tertiary education to reach their target sample size. However, raking is not feasible with empty strata and results in extreme weights when applied to data with sparsely populated strata. Therefore, we collapsed empty or sparsely populated age and education strata with adjacent strata in cases where a stratum contained less than 5% of respondents within a country.

We had to exclude 2,388 participants before raking: This was either because they had missing values for gender, age and education (raking requires participant data for all post-stratification variables) or because they identified with genders other than female or male (the World Population Prospects 2022 do not contain population margins for them). The ds_final dataset including the raked weights therefore contains only *N* = 69,534 participants, whereas the ds_main dataset without weights retains participants for whom raking was not possible and thus contains the complete valid sample of *N* = 71,922.

The raking procedures yielded the (1) post-stratification weights at country level. Next, we computed sample size weights for each country, which accounted for different sample sizes, and multiplied them with the post-stratification weights at country level to obtain the (2) post-stratification weights at global level. For weighted multilevel analyses with R’s *lme4* package^61^, we prepared (3) rescaled post-stratification weights created with the *rescale_weights()* function of the *datawizard* package (v0.10.0)^62^, which implements an algorithm proposed by Asparouhov^63^ and Carle^64^. For more details, see the R code shared with the dataset.

Combining post-hoc weighting with balanced quota sampling has several advantages: The balanced quotas help collect enough data for underrepresented and hard-to-reach participants (e.g., 18-29 y/o men in Switzerland or 50 + y/o women in Ghana). This benefits statistical analyses, whose robustness may suffer when applied to barely sampled individuals^65^. Post-hoc weighting spared us the effort to impose representative quotas and allowed us to correct sample distributions even for non-quoted demographic characteristics like education. This is important from a validity standpoint (we compensated to some degree that participant panels in some countries like India were overpopulated by higher-educated individuals, see Supplementary Table 4) and a budget perspective (imposing education quotas would have increased the duration and costs of data collection)^66^. However, these advantages come at the cost of some drawbacks, i.e. (1) exclusion of participants for which post-stratification data is not available; (2) a small number of cases with large weights in a few countries like Nigeria; (3) reduced precision in countries where quota targets had to be relaxed or where adjacent strata needed to be collapsed^65–68^. Other data collection procedures, such as probability sampling, would have compensated some of these limitations, yet they have other disadvantages, such as high costs and implementation difficulties at a global scale such as that of the TISP project^68^.

### Sample characteristics

The cleaned dataset contains 71,922 participants from 68 countries (ds_main, see *Data Records* section). Table 2 shows the characteristics of the unweighted and the weighted global samples. For sample characteristics across countries, see Tables 3–5 (weighted) and Supplementary Tables 3–5 (unweighted).

**Table 2:** Characteristics of the final sample (unweighted and weighted data).

		Unweighted	Weighted
*N*		71,992	69,534
Countries		68	68
Gender	% male	50.25	49.29
	% female	49.75	50.71
Age	*M*	43.68	45.69
	*SD*	15.08	16.49
Age groups	% 18–29 years	21.76	21.07
	% 30–39 years	21.25	18.57
	% 40–49 years	20.43	17.22
	% 50–59 years	18.96	16.64
	% 60+ years	17.60	26.51
Education	% none	0.12	0.25
	% primary	2.41	4.10
	% secondary	38.23	67.59
	% tertiary	59.24	28.06
Annual household income in USD	*Me*	19,349	16,571
	*SD*	9,363,308	6,014,711
Political orientation (conservative)	*M*	3.00	3.02
	*SD*	1.16	1.17
Political orientation (right)	*M*	3.18	3.19
	*SD*	1.07	1.09
Religiosity	*M*	2.77	2.77
	*SD*	1.40	1.41
Place of residence	% urban	73.33	70.95
	% rural	26.67	29.05

**Table 3:** Sample characteristics across countries, weighted data (1).

Country	*n*	Gender		Age		Age group				
		% female	% male	*M*	*SD*	% 18-29 years	% 30-39 years	% 40-49 years	% 50-59 years	% 60+ years
Albania	377	50.03	49.97	40.22	12.77	22.97	16.99	39.21	16.12	4.71
Argentina	509	50.46	49.54	43.05	16.11	25.76	20.10	18.08	13.73	22.33
Australia	3,560	50.36	49.64	47.12	18.07	20.78	18.95	16.37	15.53	28.37
Austria	1,076	50.78	49.22	48.04	15.99	17.01	16.62	15.90	18.87	31.59
Bangladesh	496	50.40	49.60	37.89	14.90	32.99	23.05	17.97	17.81	8.17
Belgium	2,052	50.61	49.39	48.70	17.17	17.95	16.39	16.24	17.13	32.28
Bolivia	548	49.83	50.17	36.72	12.78	34.77	23.70	17.32	22.07	2.14
Botswana	508	50.62	49.38	36.76	12.73	34.03	26.80	18.72	16.91	3.53
Brazil	1,336	50.87	49.13	42.38	15.17	25.19	21.64	18.88	15.35	18.95
Bulgaria	497	51.49	48.51	49.00	15.43	13.56	16.38	18.15	16.73	35.19
Cameroon	473	50.15	49.85	34.78	13.05	41.12	24.96	15.93	14.86	3.13
Canada	2,535	50.31	49.69	47.69	16.76	19.17	17.23	15.80	16.56	31.24
Chile	1,058	50.37	49.63	43.82	16.12	23.40	20.36	17.54	15.80	22.91
China	526	48.91	51.09	45.35	15.09	18.25	20.39	18.14	20.32	22.91
Colombia	514	50.64	49.36	41.95	15.40	28.06	21.62	17.41	15.09	17.81
Congo DR	408	50.44	49.56	35.72	13.40	42.25	22.96	14.75	10.22	9.81
Costa Rica	573	49.95	50.05	42.48	15.38	24.90	21.63	17.57	15.44	20.45
Côte d’Ivoire	514	49.47	50.53	34.97	12.58	42.00	23.45	17.47	13.75	3.34
Cyprus	509	49.92	50.08	44.55	15.24	18.57	22.80	18.50	15.45	24.68
Czech Republic	502	50.75	49.25	48.65	16.05	14.76	16.52	20.37	15.90	32.45
Denmark	1,227	50.26	49.74	48.69	17.44	19.49	14.98	15.77	17.12	32.64
Egypt	512	49.39	50.61	38.40	14.64	31.82	24.57	18.30	12.95	12.36
Ethiopia	455	49.75	50.25	31.38	10.24	42.51	23.31	25.36	8.82	0.00
Finland	1,009	50.60	49.40	49.47	17.26	17.24	16.02	14.90	15.76	36.08
France	2,029	51.66	48.34	48.66	15.66	17.09	15.39	16.25	16.51	34.77
Georgia	528	52.98	47.02	45.86	15.40	19.12	19.51	17.02	16.74	27.61
Germany	8,134	50.66	49.34	49.51	16.30	15.90	15.63	14.41	18.97	35.08
Ghana	509	50.12	49.88	34.54	11.81	36.27	25.17	33.68	0.00	4.87
Greece	1,449	51.01	48.99	48.50	15.01	15.18	14.06	18.07	17.65	35.04
Hong Kong	599	53.88	46.12	48.44	15.23	14.17	17.16	17.70	18.62	32.35
Hungary	508	52.07	47.93	47.86	15.55	16.61	15.66	19.77	15.73	32.23
India	502	48.27	51.73	40.54	17.18	30.82	22.56	17.97	13.60	15.05
Indonesia	2,104	49.64	50.36	39.81	13.51	27.21	21.75	19.99	23.98	7.08
Ireland	506	50.45	49.55	45.64	15.79	19.33	17.91	20.14	16.23	26.39
Israel	1,049	50.14	49.86	43.83	16.68	25.37	19.51	17.83	13.48	23.82
Italy	1,520	51.26	48.74	50.42	15.79	14.29	13.43	17.19	19.02	36.07
Japan	1,004	51.39	48.61	51.43	16.56	13.54	12.61	16.58	15.63	41.65
Kazakhstan	520	51.97	48.03	42.18	14.45	23.17	23.67	18.41	15.70	19.06
Kenya	513	50.43	49.57	35.59	13.55	40.17	25.05	16.46	12.54	5.78
Malaysia	1,046	48.87	51.13	40.29	15.19	28.55	24.34	17.91	13.82	15.38
Mexico	532	51.18	48.82	41.01	15.30	28.44	21.24	18.69	14.59	17.04
Morocco	503	49.61	50.39	40.47	15.35	27.47	22.17	18.75	14.74	16.87
Netherlands	1,427	50.31	49.69	47.45	16.29	18.88	15.43	15.07	17.93	32.69
New Zealand	2,028	50.44	49.56	46.98	17.90	21.37	18.23	15.98	16.41	28.02
Nicaragua	499	50.71	49.29	36.94	13.16	34.85	23.78	17.63	20.77	2.97
Nigeria	1,040	49.42	50.58	35.04	14.09	40.45	23.19	16.60	14.46	5.30
Norway	513	49.56	50.44	48.38	17.44	19.25	17.31	16.69	16.75	30.00
Peru	513	50.49	49.51	40.49	15.35	28.85	22.00	18.12	13.62	17.42
Philippines	661	49.23	50.77	39.42	14.60	33.30	22.30	17.82	13.36	13.23
Poland	3,037	51.64	48.36	47.27	16.10	15.99	19.11	18.69	14.74	31.47
Portugal	502	52.82	47.18	48.63	15.31	15.49	14.03	18.12	17.33	35.04
Romania	444	51.65	48.35	48.18	16.31	15.77	16.87	19.21	16.98	31.17
Russia	1,518	53.56	46.44	46.32	14.96	15.88	21.36	18.24	15.98	28.53
Serbia	575	52.06	47.94	47.45	15.47	16.10	16.56	18.02	16.48	32.85
Slovakia	543	51.18	48.82	47.69	15.96	16.43	18.82	19.76	15.85	29.15
Slovenia	528	49.74	50.26	48.16	15.67	14.89	16.35	18.22	17.33	33.21
South Africa	1,027	51.35	48.65	39.40	14.80	29.87	26.94	16.06	14.04	13.09
South Korea	500	50.06	49.94	47.41	15.64	17.85	15.84	18.42	19.35	28.55
Spain	1,015	50.99	49.01	48.75	15.52	15.07	14.98	19.83	18.28	31.84
Sweden	1,013	49.63	50.37	49.15	17.55	18.51	17.29	15.81	16.00	32.39
Switzerland	1,018	50.37	49.63	47.62	15.60	16.76	17.44	16.85	18.24	30.71
Taiwan	1,206	50.46	49.54	46.15	15.47	18.09	17.92	19.16	17.78	27.05
Türkiye	508	49.88	50.12	41.19	14.78	26.37	21.78	19.26	15.49	17.08
Uganda	513	50.49	49.51	33.46	13.11	48.78	23.41	8.63	19.10	0.08
Ukraine	1,020	53.72	46.28	46.36	14.86	15.35	20.18	18.08	16.50	29.90
United Kingdom	2,008	50.60	49.40	48.00	16.82	18.71	16.88	15.75	17.21	31.45
United States of America	2,580	50.46	49.54	47.33	17.48	20.48	17.51	15.98	16.47	29.56
Uruguay	325	51.56	48.44	47.20	14.37	9.08	31.74	17.05	15.13	26.99

**Table 4:** Sample characteristics across countries, weighted data (2).

Country	Education				Annual household income in USD		Political orientation (conservative)	
	% none	% primary	% secondary	% tertiary	*Me*	*SD*	*M*	*SD*
Albania	6.23	6.61	71.17	15.98	4,620	223,241	2.65	1.56
Argentina	0.00	3.40	75.27	21.33	1,013	139,767	2.69	1.06
Australia	0.17	3.05	58.91	37.87	49,693	275,791	3.31	1.18
Austria	0.00	3.72	68.20	28.07	26,779	57,149	2.77	1.01
Bangladesh	0.00	5.62	85.74	8.64	1,139	4,069	2.96	1.64
Belgium	0.00	3.69	63.62	32.68	46,915	95,721	2.94	1.01
Bolivia	0.00	2.63	73.85	23.52	616	43,269	3.06	1.48
Botswana	0.00	0.00	89.30	10.70	2,350	7,248	3.24	1.22
Brazil	0.93	37.64	47.74	13.69	341	84,877	3.34	1.46
Bulgaria	0.82	0.95	74.13	24.10	8,158	81,574	3.15	0.91
Cameroon	0.00	2.01	91.65	6.34	10	23,840	3.35	1.59
Canada	0.47	2.74	48.73	48.06	43,460	57,160	2.96	1.19
Chile	0.49	2.80	75.60	21.11	1,565	1,921,289	2.87	1.31
China	1.75	14.60	76.38	7.27	26,535	37,694	2.75	1.26
Colombia	0.00	2.79	72.89	24.32	860	7,349	2.81	1.21
Congo DR	0.00	0.00	92.12	7.88	2,000	57,844	3.00	1.57
Costa Rica	0.00	8.30	70.60	21.11	2,266	91,009	3.33	1.53
Côte d’Ivoire	1.17	1.71	92.06	5.06	1,077	20,185	3.05	1.38
Cyprus	0.33	4.32	58.95	36.40	16,079	147,730	2.73	1.00
Czech Republic	0.19	3.93	76.09	19.80	19,656	23,623	3.22	0.92
Denmark	0.01	1.61	60.04	38.34	43,875	82,863	2.82	0.88
Egypt	1.95	0.49	82.91	14.65	1,963	5,641	3.80	1.51
Ethiopia	0.82	2.57	90.71	5.90	186	152,679	2.77	1.39
Finland	0.00	20.07	44.43	35.50	40,630	51,979	2.96	1.10
France	0.21	1.25	66.79	31.75	25,488	32,230	2.98	1.06
Georgia	0.53	4.91	60.59	33.97	16,986	104,879	3.31	1.03
Germany	0.04	0.48	79.00	20.48	27,122	2,070,575	2.94	0.98
Ghana	0.00	10.17	84.83	5.00	1,569	24,602	3.07	1.40
Greece	0.22	5.22	69.17	25.39	15,519	80,425	2.74	0.98
Hong Kong	0.00	5.18	58.92	35.90	51,004	56,653	2.80	0.95
Hungary	0.00	3.46	75.65	20.89	8,412	20,699	3.04	1.11
India	0.00	7.77	79.55	12.68	4,858	11,994	3.48	1.42
Indonesia	0.45	1.05	89.08	9.42	3,234	18,403	3.44	1.04
Ireland	0.00	4.02	56.98	39.00	43,043	311,216	2.88	0.97
Israel	0.00	0.85	61.04	38.12	5,473	31,763	2.48	1.12
Italy	0.00	0.73	83.77	15.50	27,247	41,617	2.71	1.02
Japan	0.61	0.20	53.13	46.06	29,944	155,393	3.22	0.99
Kazakhstan	0.29	0.00	78.16	21.55	2,177	267,998	3.43	1.17
Kenya	0.71	2.37	88.95	7.97	1,086	7,203	3.07	1.41
Malaysia	0.34	1.75	75.06	22.84	4,541	46,693,259	3.01	0.80
Mexico	0.94	1.67	80.72	16.66	3,335	145,874	2.77	1.29
Morocco	0.67	5.69	79.47	14.16	3,361	204,008	3.87	1.26
Netherlands	0.10	3.34	66.07	30.49	44,694	163,168	2.88	0.95
New Zealand	0.33	6.38	67.09	26.20	44,915	958,007	3.34	1.13
Nicaragua	0.53	5.83	82.00	11.64	673	2,235	2.95	1.42
Nigeria	0.00	4.75	77.90	17.35	2,173	85,994	3.61	1.24
Norway	0.00	1.60	61.70	36.70	45,962	77,386	2.92	1.07
Peru	0.00	0.00	79.72	20.28	1,857	57,290	3.55	1.02
Philippines	0.84	0.76	70.14	28.26	2,674	21,033	3.41	1.15
Poland	0.03	4.76	70.81	24.40	13,477	33,383	2.90	1.23
Portugal	0.00	5.83	73.47	20.70	21,714	68,276	2.74	0.77
Romania	0.00	1.30	83.70	15.00	2,224	25,605	2.52	1.03
Russia	0.00	0.42	34.87	64.71	5,786	578,348	3.24	1.03
Serbia	0.00	2.12	79.19	18.69	1,387	161,495	2.71	1.19
Slovakia	0.00	5.74	75.36	18.90	13,787	14,832	3.28	1.10
Slovenia	0.00	4.03	72.18	23.79	12,895	16,084,963	2.63	1.21
South Africa	0.00	0.17	93.70	6.13	8,217	221,034	3.27	1.09
South Korea	0.00	2.84	50.72	46.44	36,450	37,207	3.11	0.91
Spain	0.58	15.54	51.79	32.10	22,081	151,087	2.77	1.09
Sweden	0.05	5.52	60.43	34.00	38,409	56,842	2.86	1.04
Switzerland	0.27	17.13	48.39	34.20	75,879	106,299	2.85	1.07
Taiwan	0.00	4.89	48.53	46.58	29,264	45,666	2.44	1.14
Türkiye	0.00	5.51	79.69	14.80	5,801	7,478	3.05	1.38
Uganda	0.00	0.00	94.64	5.36	1,073	24,594	3.26	1.34
Ukraine	0.21	1.32	54.13	44.35	2,721	5,780	3.09	1.32
United Kingdom	0.08	1.07	61.25	37.60	36,984	127,316	3.01	1.09
United States of America	0.56	3.63	39.54	56.26	50,000	231,087	3.22	1.35
Uruguay	0.00	1.83	86.29	11.88	1,087	136,507	2.62	1.32

**Table 5:** Sample characteristics across countries, weighted data (3).

Country	Political orientation (right)		Religiosity		Place of residence	
	*M*	*SD*	*M*	*SD*	% rural	% urban
Albania	2.95	1.06	3.73	1.16	15.03	84.97
Argentina	3.50	1.15	2.77	1.35	10.50	89.50
Australia	3.38	1.08	2.67	1.41	26.34	73.66
Austria	2.98	0.95	2.29	1.27	50.25	49.75
Bangladesh	3.79	1.29	4.02	1.13	39.95	60.05
Belgium	3.17	1.09	2.07	1.18	54.95	45.05
Bolivia	3.55	1.23	3.47	1.24	15.77	84.23
Botswana	3.29	1.14	3.77	1.31	36.26	63.74
Brazil	3.30	1.46	3.71	1.27	15.73	84.27
Bulgaria	3.23	0.88	2.99	1.19	17.91	82.09
Cameroon	3.53	1.26	3.87	1.27	20.48	79.52
Canada	3.02	1.05	2.49	1.35	26.99	73.01
Chile	3.08	1.16	2.87	1.42	14.61	85.39
China	2.69	0.96	2.02	1.24	9.98	90.02
Colombia	3.11	1.27	3.42	1.38	12.30	87.70
Congo DR	3.38	1.60	4.21	1.06	3.08	96.92
Costa Rica	3.60	1.17	3.41	1.37	39.67	60.33
Côte d’Ivoire	3.07	1.20	4.27	1.13	20.76	79.24
Cyprus	3.16	0.87	3.28	1.24	11.35	88.65
Czech Republic	3.33	0.96	1.89	1.22	23.71	76.29
Denmark	3.05	1.05	2.19	1.15	28.18	71.82
Egypt	4.05	1.25	4.21	0.93	9.47	90.53
Ethiopia	3.17	1.25	3.85	1.23	28.14	71.86
Finland	3.17	1.09	2.25	1.23	23.93	76.07
France	3.17	1.20	2.01	1.17	51.74	48.26
Georgia	3.21	1.10	3.15	1.30	11.75	88.25
Germany	2.95	0.84	2.10	1.24	43.68	56.32
Ghana	3.50	1.22	4.05	1.21	26.33	73.67
Greece	3.06	0.83	3.14	1.29	13.93	86.07
Hong Kong	3.08	0.72	2.11	1.32	2.30	97.70
Hungary	3.12	1.11	2.27	1.23	31.43	68.57
India	3.49	1.27	3.79	1.04	32.18	67.82
Indonesia	3.54	0.90	3.74	0.84	24.29	75.71
Ireland	2.96	1.01	2.55	1.26	39.79	60.21
Israel	3.48	0.95	2.23	1.25	16.26	83.74
Italy	3.04	1.12	2.75	1.30	30.33	69.67
Japan	3.27	0.84	2.61	1.16	54.16	45.84
Kazakhstan	3.33	0.92	2.90	1.09	16.61	83.39
Kenya	3.55	1.14	4.20	1.06	26.42	73.58
Malaysia	3.16	0.71	3.82	1.05	24.10	75.90
Mexico	3.02	1.16	3.04	1.23	17.16	82.84
Morocco	3.47	1.00	3.69	1.01	13.00	87.00
Netherlands	3.16	1.08	1.99	1.28	45.15	54.85
New Zealand	3.37	1.08	2.71	1.45	23.31	76.69
Nicaragua	2.88	1.35	3.53	1.25	27.23	72.77
Nigeria	3.51	1.13	3.92	1.20	27.73	72.27
Norway	3.06	1.13	2.14	1.25	44.62	55.38
Peru	3.54	0.98	3.23	1.10	10.97	89.03
Philippines	3.63	0.99	3.59	1.15	41.33	58.67
Poland	3.14	1.20	2.82	1.27	23.20	76.80
Portugal	2.87	0.91	2.39	1.09	26.18	73.82
Romania	3.17	1.09	2.34	1.27	21.99	78.01
Russia	3.12	0.88	2.57	1.16	12.93	87.07
Serbia	2.80	0.99	3.19	1.24	21.20	78.80
Slovakia	2.98	1.07	2.97	1.33	35.40	64.60
Slovenia	2.86	1.11	2.51	1.37	34.59	65.41
South Africa	3.36	1.09	3.69	1.33	14.80	85.20
South Korea	3.14	0.99	2.27	1.35	10.06	89.94
Spain	2.86	1.12	2.33	1.27	20.22	79.78
Sweden	3.15	1.16	1.84	1.12	30.20	69.80
Switzerland	3.14	1.03	2.24	1.24	54.48	45.52
Taiwan	3.19	0.68	2.97	1.26	21.48	78.52
Türkiye	2.98	1.43	3.41	1.17	7.05	92.95
Uganda	3.97	1.20	4.38	0.99	13.03	68.97
Ukraine	3.36	1.15	2.93	1.20	20.27	79.73
United Kingdom	2.99	1.03	2.07	1.24	33.03	66.97
United States of America	3.44	1.26	3.28	1.43	35.69	64.31
Uruguay	2.83	1.37	2.19	1.34	9.87	90.13

## Data Records

The TISP dataset is available at a dedicated OSF repository: https://osf.io/5c3qd^51^. The repository includes a wiki with detailed instructions for users and contains the following folders:

- 01_data includes three versions of the TISP dataset and respondent ID data for duplicate checks (./survey-data), demographic data of target populations for computing the post-stratification weights (./population-data) and conversion rates for transforming local currencies to USD (./currency-data).
- 02_code includes R code for replicating the data pre-processing procedures and the validation analyses (see *Technical Validation* section).
- 03_models includes pre-computed *lavaan* models^69^ used in the validation analyses and a *svydesign* object^54^ of the analysis-ready dataset, all in .rds format.
- 04_figures includes all figures in high resolution.
- 05_survey-materials includes all survey materials, i.e. the questionnaires, guides, manuals and templates.
- 06_irb-documents includes the official documents certifying ethical approval from the Area Committee on the Use of Human Subjects at Harvard University as well as materials for collaborators in case they needed to seek IRB approval.

Other studies have already used the TISP dataset. For example, Cologna *et al*.^31^ used it for a global analysis of public trust in scientists. However, they included only a small subset of variables, whereas the TISP dataset contains several more measures. They conducted comprehensive descriptive and multivariate analyses to test pre-registered research questions and hypotheses, which are far beyond the scope of the current article; we only present an overview of the sample characteristics (see *Methods* section) and psychometric properties of select measures (see *Technical Validation* section).

### The datasets

The 01_data folder in the OSF repository includes three versions of the TISP dataset^51^. It contains (1) the raw dataset before any cleaning and transformations (*N* = 167,101, filename ds_full), (2) the cleaned dataset without weights (*N* = 71,922, filename ds_main), and (3) an analysis-ready dataset that includes the post-stratification weights (*N* = 69,534, filename ds_final). See the *Methods* section and Fig. 4 for the data pre-processing procedures used to prepare these datasets.

We share each of the datasets in .rds, .sav, and .csv formats. It is recommended to use the .rds files where response values are labelled. The .csv files are semicolon-delimited and use UTF-8 encoding with a Bit Order Mark (BOM), so they can be imported into Microsoft Excel, for example, with correct encoding of non-ASCII characters (missing values coded as “NA”). Open-ended answers (see *Methods* section) are provided in the languages in which they were recorded, so that users of the TISP dataset can analyse raw answers and employ translation software or services of their choice.

Researchers who wish to conduct statistical analyses that estimate parameters that are representative for target populations in terms of gender, age and education and have correct variances and standard errors should use the analysis-ready dataset. It contains three kinds of post-stratification weights (see *Methods* section and Fig. 4).

1. WEIGHT_CNTRY: This variable contains the post-stratification weights at country level, to be used for weighted analyses with single country samples.
2. WEIGHT_GLOBL: This variable contains the post-stratification weights at global level, to be used for weighted analyses with the entire analysis-ready dataset.
3. WEIGHT_MLVLM: This variable contains the rescaled post-stratification weights for weighted multilevel analyses with R’s *lme4* package^61^. Note that *svydesign* objects, which R users might prefer, cannot be included in multilevel modelling by means of R’s *survey* package v4.4-2^54^.

Using the post-stratification weights at country and global level will give point estimates (e.g., mean values, regression coefficients, etc.) that are representative in terms of gender, age and education. To obtain correct variances and standard errors of point estimates, one should use either a *svydesign* object created with the *svydesign()* function of R’s *survey* package^54^ or the rescaled post-stratification weights. We pre-computed a *svydesign* object of the TISP dataset, which can be found in the repository (folder 03_models) or reproduced by users with the R code provided.

### Survey materials

The materials available at the OSF repository also include all survey materials: the TISP core questionnaire in English, all 88 local questionnaires, the Qualtrics file in .qsf format and instructions for collaborators (data collection manual, data submission guide and the TISP guidebook).

### IRB documents

We also share the documents certifying ethical approval from the Area Committee on the Use of Human Subjects at Harvard University as well as template materials prepared for local IRB applications.

## Technical Validation

We employed several procedures to assure the validity of the TISP dataset. The survey used questions and scales that were based on established conceptual models and were validated in multiple prior studies^23–25,35,44,47^. It included attention checks to reduce satisficing and straight-lining, i.e. common problems of survey studies^70^, and was designed with an international advisory board of nine experts on public opinion and communication about science, environmental psychology, the history and sociology of science and survey methods. To enhance the invariance of questionnaire performance across countries and languages, we drew on cross-checked translations by local collaborators who were native speakers and familiar with the research topic and study context. To ensure the integrity of the data collection process, the project leads pre-registered sample size rationales and data pre-processing steps before fielding surveys, obtained ethical approval from multiple IRBs, provided templates, guides, tutorials and 1-on-1 assistance to collaborators, and required all co-authors to sign an ethical agreement. The entire TISP consortium, including the advisory board, was also involved in internal peer review of project outputs. An independent data scientist as well as TISP collaborators highly proficient in statistical analyses also reviewed the statistical code for preparing the dataset and verifying its reliability.

We took three further measures to validate the quality of the TISP dataset as detailed below. (1) We conducted a pre-test prior to the main survey to validate the measures used in the questionnaire. (2) We inspected if the attention checks had similar performance across countries and confirmed that they filtered demographic groups of respondents known to be less attentive to surveys. (3) We assessed the internal consistency, factor structures, measurement invariance and convergent validity of all four scales that we adopted from prior research or, in the case of the 12-item scale measuring trust in scientists, developed for the purpose of the TISP study.

### Pre-test

A pre-test with *N* = 401 participants was conducted in the United States in October 2022. Average completion time was 14 minutes. The questionnaire was slightly modified to improve the comprehensibility of questions and the survey flow, and two questions were added to the final questionnaire. Pre-test data are not included in the datasets presented in this article, but are available at https://osf.io/wj34h.

### Attention check performance

The questionnaire contained two attention checks (see *Methods* section). 4% of respondents who reached the first attention check did not pass it. 24% of participants who reached the second attention check did not pass it. This indicates that both attention checks – particularly the second – clearly increased data quality: They filtered numerous respondents who were likely too inattentive to provide meaningful data and might thus have compromised the reliability of the TISP data.

The attention checks also harmonized data quality across countries and polling companies. This was necessary as respondents from Brazil, India or Türkiye often failed them, whereas participants from Romania, Uruguay or the United Kingdom had much higher baseline attentiveness levels (see Supplementary Table 6).

We also validated the performance of the attention checks by verifying that they filtered respondents who are typically prone to fail such checks, i.e. people who are younger, male and lower educated^43^. To do so, we fitted logistic multilevel regression models with random intercepts across countries which predicted failing with age, gender and education, i.e. the three demographic characteristics that were measured before the first attention check and were therefore available for all participants. Unstandardised and standardised regression estimates (within-country scaled predictors) show that failing the first attention check was marginally more likely if participants were younger (*b* = −0.004, β = −0.055, *OR* = 0.946, *SE* = 0.015, *z* = −3.606, *p* < 0.001) and clearly more likely if they had no tertiary education (*b* = −0.478, β = −0.224, OR = 0.798, SE = 0.015, *z* = −14.931, *p* < 0.001). Gender was also related to failing, with males being slightly more likely to fail the first attention check than females (*b* = 0.008, β = 0.134, OR = 1.143, SE = 0.008, *z* = 17.001, *p* < 0.001). Failing the second attention check was more likely among participants who are male (*b* = 0.010, β = 0.132, *OR* = 1.141, *SE* = 0.007, *z* = 20.304, *p* < 0.001), younger (*b* = −0.031, β = −0.432, *OR* = 0.649, *SE* = 0.008, *z* = −52.292, *p* < 0.001) and lower educated, with participants who completed tertiary education being more attentive than participants who completed only primary or secondary education (*b* = −0.295, β = −0.139, *SE* = 0.007, *OR* = 0.870, *z* = −18.255, *p* < 0.001). These results indicate that the attention checks worked well and allowed us to collect similarly informative data across different demographic groups.

### Scale validations

We tested the psychometric properties and measurement performance of the 12-item scale of trust in scientists^23^, the 8-item scale of science-related populist attitudes^24^, the 3-item scale of outspokenness about science^25^, and the 4-item scale of SDO^26^, so as to provide users of the TISP dataset with information about their validity. These tests included (a) internal consistency estimates and comparisons with consistency estimates from previous research, (b) assessments of the dimensional structures via parallel analysis, exploratory factor analysis (EFA) and multi-group exploratory structural equation modelling (ESEM)^71^, (c) measurement invariance tests via confirmatory factor analysis (CFA) and (d) convergent validity analyses.

#### Perceived trustworthiness of scientists

The 12-item scale measuring perceived trustworthiness of scientists may be aggregated to a single score by computing the arithmetic mean of all response values for each respondent, with higher values indicating higher perceived trustworthiness (weighted *M* = 3.62, *SD* = 0.70, range: 1 – 5; see R code for *M* and *SD* across countries). Overall, the scale shows excellent internal consistency, captures the four trustworthiness dimensions rather distinctively, exhibits acceptable measurement performance in the global sample but limited invariance across countries and has high convergent validity.

##### Internal consistency

Scale consistency in the global sample was excellent, with Cronbach’s α = 0.93 and ω = 0.95. Such high estimates seem typical for this measure: Our pre-test survey showed values of α = 0.95 and ω = 0.96, and previous studies using similar scales like the METI^45^ also found high very estimates of α = 0.94^72^ and α = 0.95^73^. This suggests that some scale items may be somewhat redundant^74^ in some countries like the United States (estimates across countries can be replicated with the R code available at our OSF repository). However, shortening the scale, which is a preferred solution for item redundancy^74^, was no option for us, as we would not want to risk a loss of scale reliability in countries with lower estimates (e.g., Czech Republic, where α = 0.87 and ω = 0.91). Moreover, we sought to maintain sufficient subscale consistency – which had likely been reduced had we removed items from the scale – so as to accommodate dataset users who wish to analyse single trustworthiness dimensions.

##### Dimensional structure

Mardia’s test showed that multivariate normality could not be assumed (Mardia skewness = 16,773, Mardia kurtosis = 256, *p* < 0.001). Therefore, the parallel analysis and the EFA used principial axis factoring (PA) instead of maximum likelihood factoring (ML), as PA factoring outperforms ML factoring when the normality assumption is violated^75^. Polychoric parallel analysis did not find the four dimensions competence, integrity, benevolence and openness, but suggested five factors. However, EFA results showed that the items formed plausible factors that largely correspond with those four dimensions – even if there were a few cross-loadings due to which the benevolence and openness dimensions were less distinct (see Supplementary Table 7). A multilevel EFA model implemented via multi-group ESEM^71^ had good fit (*χ*² = 7,421, df = 3,433, *p* < 0.001; CFI = 0.983, TLI = 0.978, RMSEA = 0.043, SRMR = 0.039).

##### Measurement invariance

CFA that tested a model with four latent factors, each predicting its three corresponding items, indicated moderate model fit (*χ*² = 5,840, df = 48, *p* < 0.001; CFI = 0.971, TLI = 0.960, RMSEA = 0.053, SRMR = 0.025). Multi-group CFAs yielded slightly worse results (*χ*² = 12,188, df = 3,264, *p* < 0.001; CFI = 0.962, TLI = 0.948, RMSEA = 0.066, SRMR = 0.031 for the configural model). They suggested that we can assume configural invariance for the trustworthiness scale across countries, but not metric or scalar invariance (*p* < 0.001), which is typical for multi-country models.

##### Convergent validity

We tested the convergent validity of the scale by assessing zero-order correlations of the arithmetic mean of all twelve items with other constructs that were included in the TISP survey, are conceptually related to trust in scientists and were found to be associated with it in prior research: Trustworthiness was negatively related with perceptions that scientists are biased by personal and third interests, which is in line with existing findings^76^ (see Table 6). Plausibly, we also found substantial positive correlations of the trustworthiness score and confidence that scientists act in the best interests of the public^77^, willingness to be vulnerable to scientists^23^ and the belief that scientific results should be integrated into policy-making^78^. This demonstrates high convergent validity of the trustworthiness measure.

**Table 6:** Convergent validity tests: Zero-order correlations of trust in scientists, science-related populist attitudes, outspokenness about science, SDO and related constructs.

Trust in scientists^a^	Perceived bias by personal/third interests^b^	Confidence in scientists^c^	Willingness to be vulnerable^d^	Preference for evidence-informed policy-making^e^
Science-related populist attitudes^f^	−0.337 (0.004)***	0.707 (0.002)***	0.458 (0.004)***	0.293 (0.004)***
	*Perceived integrity of scientists*^g^	*Trust in scientific methods*^h^	*Right-leaning political orientation*^i^	*Social Dominance Orientation*^j^
Outspokenness about science^k^	−0.130 (0.005)***	−0.146 (0.004)***	0.176 (0.005)***	0.201 (0.004)***
	*Communicate with others about science*^l^	*Exposure to science information in messaging apps*^m^		*Talk about science with friends or family*^n^
Social Dominance Orientation^j^	0.261 (0.003)***	0.164 (0.004)***		0.205 (0.004)***
	*Right-leaning political orientation*^i^	*Conservative political orientation*°	*Support for research on defence and military technology*^p^	*Endorsement of taxes on carbon intense foods*^q^
	0.218 (0.004)***	0.194 (0.004)***	0.083 (0.004)***	0.021 (0.004)***

*Note*: Table displays weighted estimates of Pearson correlations in the analysis-ready dataset^51^ (*N* = 69,534) and bootstrapped standard errors in brackets. ***p < 0.001.

^a^Mean of the 12-item trustworthiness scale.

^b^Average agreement with the items “Scientists are only interested in their own advantage” and “Scientists are in cahoots with politicians and businesses”, i.e. the conceptions of the academic elite dimension of the SciPop Scale (1 = strongly disagree, 5 = strongly agree)^24^.

^c^Response to the item “How much confidence do you have in scientists to act in the best interests of the public?” (1 = no confidence at all, 5 = a great deal of confidence).

^d^Average response to the three items measuring willingness to be vulnerable to scientists (1 = not willing at all, 5 = very much willing).

^e^Agreement with the item “Scientists should work closely with politicians to integrate scientific results into policy-making” (1 = strongly disagree, 5 = strongly agree).

^f^Goertz score of the SciPop Scale.

^g^Average response to the three items measuring the perceived integrity of scientists, i.e. “How honest or dishonest are most scientists?”, “How ethical or unethical are most scientists?” and “How sincere or insincere are most scientists?” (1 = no integrity, 5 = very high integrity).

^h^Agreement with the item “Scientific research methods are the best way to find out if something is true or false.” (1 = strongly disagree, 5 = strongly agree).

^i^Response to the item “Please indicate your political orientation” (1 = strongly left-leaning, 5 = strongly right-leaning).

^j^Mean of the 4-item scale measuring social dominance orientation.

^k^Mean of the 3-item outspokenness scale.

^l^Average agreement with the four items measuring how often respondents communicate with others about science (1 = never, 7 = once or more per day).

^m^Response to the item “Over the past 12 months, how often have you come across information about science in instant messaging conversations with friends or family (e.g., WhatsApp, Line, Telegram)?” (1 = never, 7 = once or more per day).

^n^Response to the item “Over the past 12 months, how often have you come across information about science in conversations with friends or family (i.e., outside the Internet and messaging apps)?” (1 = never, 7 = once or more per day).

°Response to the item “Please indicate your political orientation” (1 = strongly liberal, 5 = strongly conservative).

^p^Response to the item “What goals should scientists prioritize? – Developing defence and military technology” (1 = very low priority, 5 = very high priority).

^q^Response to the item “Please indicate your level of support for the following policies. – Increasing taxes on carbon intense foods (e.g., beef and dairy products)” (1 = not at all, 2 = moderately, 3 = very much).

#### Science-related populist attitudes

There are different ways to aggregate responses to the eight items of the SciPop Scale^24^ into a single score that indicates affinity or opposition to science-related populism, such as taking the average of all response values (“Bollen approach”) or classifying participants as populist vs. non-populist based on their responses (“Sartori approach”)^79^. The authors of the SciPop Scale recommend the “Goertz approach”^80^. This approach suggests that the smallest of the four dimension scores determines someone’s net support for science-related populism, regardless of the magnitude of the other three dimension scores. It accounts for the conceptual premise that all components of science-related populism have to be concurrently present within a person to diagnose science-related populist attitudes, whereas the absence of one or more components would disqualify someone to be classified as a proponent of science-related populism (see Mede *et al*.^79^ and Wuttke *et al*.^80^ for more details). The Goertz approach has thus become a preferred procedure in research on both science-related and political populism^11,81–83^. We therefore applied this approach when assessing the psychometric properties and measurement performance of the SciPop Scale in the TISP dataset: First, we calculated unweighted arithmetic means of the response values for each of the four 2-item components of the scale (see *Methods* section). Second, we took the lowest of these four means as an indicator of someone’s overall support for science-related populism (weighted *M* = 2.32, *SD* = 0.91), with higher values indicating stronger support (range: 1 – 5). In sum, our validity tests indicate high internal consistency of the SciPop Scale, confirm the four-dimensional factor structure, demonstrate good performance in the global sample despite somewhat limited measurement invariance and suggest sufficient convergent validity.

##### Internal consistency

The internal consistency of the SciPop Scale was fairly high (α = 0.79 and ω = 0.87). Reliability estimates were within the range estimates in previous studies, which find values of α = 0.75 in Taiwan and up to α = 0.90 in Austria, for example^84^.

##### Factor structure

Polychoric parallel analysis confirmed the four-dimensional conceptualisation of the SciPop Scale. Oblique polychoric EFA showed that the eight items formed four plausible factors that correspond with the four conceptual dimensions of science-related populist attitudes (see Supplementary Table 8). Mardia’s test showed that multivariate normality of the SciPop Scale could not be assumed (Mardia skewness = 3,992, Mardia kurtosis = 122, *p* < 0.001), so the parallel analysis and the EFA used PA factoring instead of ML factoring. An ESEM-based multilevel EFA model had excellent fit (*χ*² = 1,845, df = 1,208, *p* < 0.001; CFI = 0.992, TLI = 0.987, RMSEA = 0.029, SRMR = 0.026).

##### Measurement invariance

A CFA model with four second-order factors, each predicting its two corresponding items, and one first-order factor had satisfactory fit (*χ*² = 1,449, df = 16, *p* < 0.001; CFI = 0.976, TLI = 0.958, RMSEA = 0.046, SRMR = 0.033). Multi-group CFAs showed similar results (*χ*² = 3,510, df = 1,088, *p* < 0.001; CFI = 0.968, TLI = 0.944, RMSEA = 0.060, SRMR = 0.037 for the configural model). They suggested that we can not assume metric or scalar invariance across countries (*p* < 0.001).

##### Convergent validity

The SciPop Scale exhibits sufficient convergent validity: We found significant negative correlations of science-related populist attitudes with the extent to which participants perceive scientists to have integrity (see Table 6), replicating recent findings^85^. Support for science-related populism was also negatively associated with trust in scientific methods^24^. We found significant positive correlations with right-leaning political orientation and SDO, which corresponds with prior research^79,86^.

#### Outspokenness about science

The 3-item scale measuring outspokenness about science may be aggregated to a single score by computing the arithmetic mean of the response values for each respondent, with higher values indicating higher outspokenness (weighted *M* = 3.87, *SD* = 0.98, range: 1 – 5). The psychometric tests indicate strong internal consistency, unidimensionality, mediocre measurement invariance and good convergent validity.

##### Internal consistency

We find that the outspokenness scale has very high internal consistency in our sample, with α = 0.89 and ω = 0.89. This is within the range of estimates in previous studies, which report values between α = 0.79^87^ and α = 0.95^25^ for slightly different versions of the scale.

##### Factor structure

We confirmed the unidimensionality of the scale using polychoric parallel analysis, which showed that all three items load on one common factor. Multivariate normality could not be assumed (Mardia skewness = 7,522, Mardia kurtosis = 171, *p* < 0.001), so the parallel analyses relied on PA factoring. An ESEM-based multilevel EFA model had very good fit (*χ*² = 232, df = 135, *p* < 0.001; CFI = 0.997, TLI = 0.995, RMSEA = 0.034, SRMR = 0.019).

##### Measurement invariance

CFA indicated mixed results: Some indicators indicated that a one-factor model had good fit according to common rules of thumb^88^, but others did not (*χ*² = 347, df = 1, *p* < 0.001; CFI = 0.985, TLI = 0.955, RMSEA = 0.090, SRMR = 0.112). This is perhaps partly because we had to fix the variance of the latent factor to 1, otherwise the model would have been saturated with df = 0. Multi-group CFAs showed even less ideal results (*χ*² = 597, df = 68, *p* < 0.001; CFI = 0.981, TLI = 0.944, RMSEA = 0.111, SRMR = 0.112 for the configural model). They indicated that we can not assume metric or scalar invariance (*p* < 0.001).

##### Convergent validity

Correlations of the outspokenness scale and other constructs measured in the TISP survey are consistent with previous studies, which confirms its convergent validity: Outspokenness was positively associated with communicating with others about science^25^, exposure to science infomation in messaging apps^81^ and having conversations about science with friends or family outside the Internet^89^ (see Table 6).

#### Social dominance orientation

The 4-item scale measuring SDO may be aggregated by computing the arithmetic mean of the response values for each respondent, with higher values indicating stronger SDO (weighted *M* = 3.62, *SD* = 1.76, range: 1 – 10). The psychometric tests indicate mediocre internal consistency, ambiguous results regarding the dimensionality and low measurement invariance, but satisfactory convergent validity.

##### Internal consistency

The SDO scale exhibits mediocre consistency in the TISP dataset (α = 0.57 and ω = 0.59). However, relatively low estimates like these are common for this scale: Previous comparative research found estimates as small as α = 0.34 in Türkiye, α = 0.44 in Lebanon, α = 0.48 in Taiwan and Indonesia, α = 0.52 in Serbia and South Africa and α = 0.53 in the Netherlands^26^. Further studies suggest slightly better reliability in countries like Austria, the Czech Republic, Germany, France, Hungary, Italy and Poland, where estimates ranged from α = 0.69 to α = 0.74 and ω = 0.62 to 0.76^90^. This is largely in line with what we find for these countries.

##### Factor structure

Dimensionality tests of the SDO scale gave mixed results: The Kaiser criterion suggested unidimensionality as we find one factor with an eigenvalue greater than one^91^. This replicates previous findings^90^. However, parallel analysis and EFA based on Pearson correlations indicate two factors, with the two reverse-worded items loading on the first factor and the two non-reversed items loading on the second factor, which has been described as a common methodological artifact^92^. The parallel analysis and the EFA used PA factoring since multivariate normality could not be assumed (Mardia skewness = 13,278, Mardia kurtosis = 49, *p* < 0.001). An ESEM-based multilevel EFA model had bad fit, which is likely due to the somewhat ambiguous factor structure and corresponds with the mediocre reliability of the scale (*χ*² = 7172, df = 333, *p* < 0.001; CFI = 0.644, TLI = 0.570, RMSEA = 0.181, SRMR = 0.097).

##### Measurement invariance

A one-factor CFA model did not have good fit (*χ*² = 4,075, df = 3, *p* < 0.001; CFI = 0.728, TLI = 0.455, RMSEA = 0.179, SRMR = 0.095). We needed to constrain the variance of the item “We should not push for group equality” to 1 in order to avoid Heywood cases^93^. Multi-group CFA models also showed bad fit (*χ*² = 6,310, df = 201, *p* < 0.001; CFI = 0.682, TLI = 0.363, RMSEA = 0.220, SRMR = 0.083 for the configural model). One can not assume metric or scalar measurement invariance across countries (*p* < 0.001), which is conform to findings of previous research^90^. The poor fit of the one-factor models is likely a result of the unstable factor structure of the SDO scale in the TISP dataset. A two-factor CFA model performs clearly better (*χ*² = 889, df = 2, *p* < 0.001; CFI = 0.941, TLI = 0.822, RMSEA = 0.103, SRMR = 0.034), but fitting two-factor multi-group CFAs failed due to Heywood cases and non-identification.

##### Convergent validity

Consistent with extant findings, we find positive correlations of SDO with right-leaning political orientation^90^, conservativism^94^, support for research on developing defence and military technology^95^, and opposition to laissez-faire capitalism, here operationalised as endorsement of taxes on carbon intense foods^96^ (see Table 6). This is evidence that the SDO scale has sufficient convergent validity.

## Usage Notes

### Online repository

The TISP dataset, additional data, R code, pre-computed statistical models, additional materials and high-resolution versions of the figures presented in this article are available at the OSF: https://osf.io/5c3qd (see *Data Records* section)^51^. The datasets are ready to use with popular statistical software like R (recommended), IBM SPSS Statistics and Microsoft Excel.

The OSF repository contains a wiki with information on the content of the folders and further instruction on how to use the files. The R code accompanying the datasets (folder 02_code) includes detailed annotations so that users can easily retrace and replicate the data-preprocessing procedures and validation analyses.

### Online dashboard

We developed a web-based data visualisation dashboard using R shiny^97^. Users may explore data on key variables of the TISP project across countries and subsamples. The dashboard is under development. It can be accessed at: https://tisp.shinyapps.io/TISP/.

## Supplementary information

Supplementary Tables and Figures

## Data Availability

All data as well as the R code, and pre-computed models underlying the analyses described in this article, and Figs. 1–4 in high resolution are available at the Open Science Framework: https://osf.io/5c3qd.
