# Supplementary material for: Perceptions of science, science communication, and climate change attitudes in 68 countries – the TISP dataset
Source: Sci Data. 2025 Jan 20;12:114. doi: 10.1038/s41597-024-04100-7 (PMC11747281; doi:10.1038/s41597-024-04100-7)
Supplement: Supplementary file 1 — Supplementary Tables and Figures [file 41597_2024_4100_MOESM1_ESM.docx]

Supplementary Information to

**Perceptions of science, science communication, and climate change attitudes in 68 countries – the TISP dataset**

***Supplementary Tables***

**Supplementary Table 1. Overview IRB applications for ethics approval**

| Country of data collection | Surname lead collaborator(s) | Partner institution | Outcome / exception | IRB reference |
| --- | --- | --- | --- | --- |
| Albania | Bajrami | University of Tirana | Harvard IRB approval sufficient | n/a |
| Argentina | Nobre | Universidade do Estado de Minas Gerais | Harvard IRB approval sufficient | n/a |
| Australia | Stanley | Australian National University | IRB approval granted | 2022 506 |
| Austria | Lamm | University of Vienna | IRB approval granted | EK Nr: 000412 |
| Bangladesh | Islam | Jahangirnagar University | Harvard IRB approval sufficient | n/a |
| Belgium | De Peuter | KU Leuven | IRB approval granted | G-2022-5777-R2(MAR) |
| Bolivia | Cologna | Harvard University | Harvard IRB approval sufficient | n/a |
| Botswana | Kotcher | George Mason University | Exempt from full IRB review | IRBNet #2008429 |
|  | Bati | Health Research and Development Division, Ministry of Health, Botswana | IRB approval granted | HPRD: 6/14/1 |
| Brazil | Azevedo | Friedrich Schiller University Jena | Harvard IRB approval sufficient | n/a |
| Bulgaria | Lamm | University of Vienna | IRB approval granted | EK Nr: 000412 |
| Cameroon | Ntui-Njock | University of Buea | IRB approval granted | 2196-11 |
| Canada | Palmer-Hague | Trinity Western University | Harvard IRB approval sufficient | n/a |
|  | Breeden | Pomona College | Exempt from full IRB review | #23-01-04 |
|  | Goddard | University of Alberta | IRB approval granted | Pro00128481 |
| Chile | Garrido-Vásquez | Universidad de Concepción | IRB approval granted | CEBB 1372-2022 |
| China | Xia | Tongji University | Harvard IRB approval sufficient | n/a |
| Colombia | Douglas | University of Kent | IRB approval granted | 8016 |
| Congo DR | Späth | Université Officielle de Bukavu | IRB approval granted | UOB/FSSPA/CFE/05/2023 |
| Costa Rica | Altenmüller | LMU Munich | No IRB application required as per national/institutional regulations | n/a |
| Côte d’Ivoire | Toko | Mohammed VI Polytechnic University | IRB approval granted | n/a |
| Cyprus | Kyza | Cyprus University of Technology | Harvard IRB approval sufficient | n/a |
| Czech Republic | Klabíková Rábová | Charles University | Harvard IRB approval sufficient | n/a |
| Denmark | Fuglsang | Aarhus University | Harvard IRB approval sufficient | n/a |
| Egypt | Aldoh | University of Amsterdam | IRB approval granted | FMG-1067 |
| Ethiopia | Debnath | University of Cambridge | Exempt from full IRB review | 5051.145 |
| Finland | Koivula | University of Turku | No IRB application required as per national/institutional regulations | n/a |
| France | Bret | Université Paris Nanterre (CER-PN) | IRB approval granted | n/a |
| Georgia | Pisareva | KIMEP University | Harvard IRB approval sufficient | n/a |
| Germany | Von Bubnoff | Rhine-Waal University | No IRB application required as per national/institutional regulations | n/a |
|  | Kojan | University of Lübeck | IRB approval granted | 2022-632 |
|  | Dries | Harding Center for Risk Literacy, University of Potsdam | Harvard IRB approval sufficient | n/a |
|  | Genschow | Leuphana University Lüneburg | Harvard IRB approval sufficient | n/a |
|  | Fischer | Leibniz Institut für Wissensmedien | Declared exempt from full IRB review | n/a |
|  | Metag | University of Münster | Harvard IRB approval sufficient | n/a |
|  | Rödder | Universität Hamburg | Harvard IRB approval sufficient | n/a |
|  | Dohle | University of Bonn | No IRB application required as per national/institutional regulations | n/a |
| Ghana | Feuerriegel | LMU Munich | Harvard IRB approval sufficient | n/a |
| Greece | Gkinopoulos | University of Warsaw | IRB approval granted | n/a |
|  | Kuppens | University of Groningen | Declared exempt from full IRB review | PSY-2223-S-0167 |
| Hong Kong | Huang | City University of Hong Kong | IRB approval granted | HU-STA-00000276 |
| Hungary | Szaszi | Eötvös Loránd University | IRB approval granted | 2022/472 |
| India | Debnath | University of Cambridge | Declared exempt from full IRB review | 5051.145 |
| Indonesia | Gordon | Sunan Kalijaga State Islamic University | IRB approval granted | B-292a/Un.02/L3/TL/01/2023 |
|  | Facciani | University of Notre Dame | No IRB application required as per national/institutional regulations | n/a |
| Ireland | Roche | Trinity College Dublin | IRB approval granted | n/a |
| Israel | Herziger | Technion – Israel Institute of Technology | IRB approval granted | 135260 |
| Italy | Bertsou | University of St. Gallen | IRB approval granted | HSG-EC-020221101 |
| Japan | Tanaka | Waseda University | IRB approval granted | #2022-446 |
| Kazakhstan | Ibadildin | KIMEP University | IRB approval granted | n/a |
| Kenya | Amollo | National Commission for Science, Innovation and Technology | IRB approval granted | EUISERC/APP/228/2023 |
|  |  | Egerton University | IRB approval granted | EUISERC/APP/244/2023 |
| Malaysia | Facciani | University of Notre Dame | No IRB application required as per national/institutional regulations | n/a |
| Mexico | Hernández-Mondragón | Center for Research and Advanced Studies of the National Polytechnic Institute | Harvard IRB approval sufficient | n/a |
| Morocco | Jeddi | Mohammed VI Polytechnic University | IRB approval granted | n/a |
| Netherlands | Kuppens | University of Groningen | Declared exempt from full IRB review | PSY-2223-S-0167 |
| New Zealand | Stanley | Australian National University | IRB approval granted | 2022 506 |
|  | Milfont | University of Waikato | IRB approval granted | FS2022-64 |
| Nicaragua | Röer | Witten/Herdecke University | Harvard IRB approval sufficient | n/a |
| Nigeria | Von Bubnoff | Rhine-Waal University | Declared exempt from full IRB review | n/a |
| Norway | Kristiansen | University of Bergen | Harvard IRB approval sufficient | n/a |
| Peru | Monge | Ernest Manheim Public Opinion Laboratory | IRB approval granted | D20230104 |
| Philippines | Cases | College of Social Sciences and Philosophy, University of the Philippines Diliman | IRB approval granted | CSSPERB-2023-005 |
| Poland | Hensel | University of Warsaw | IRB approval granted | n/a |
|  | Czarnek | Jagiellonian University | IRB approval granted | KE/1_2023 |
| Portugal | Santos | Institute of Environmental Health Lisbon of the School of Medicine | Harvard IRB approval sufficient | n/a |
| Romania | Etienne | VU University Amsterdam | Declared exempt from full IRB review | 2023-1-30-434 |
| Russia | Grigoryev | HSE University | IRB approval granted | 01/10.2022 |
| Serbia | Lazić | University of Belgrade | IRB approval granted | #2022-76 |
| Slovakia | Buchel | Institute for Sociology of the Slovak Academy of Sciences | IRB approval granted | IRB22-1046 |
| Slovenia | Muršič | University of Ljubljana | Harvard IRB approval sufficient | n/a |
| South Africa | Joubert | Stellenbosch University | IRB approval granted | SU - SEBR - 26177 |
| South Korea | Scoggins | KIMEP University | Harvard IRB approval sufficient | n/a |
| Spain | Cabrera Alvarez | University of Essex | Harvard IRB approval sufficient | n/a |
| Sweden | Parnamets | Karolinska Institutet | No IRB application required as per national/institutional regulations | n/a |
| Switzerland | Freundt | University of Fribourg | Harvard IRB approval sufficient | n/a |
| Taiwan | Rauchfleisch | National Taiwan University | IRB approval granted | 202312HS006 |
| Türkiye | Çoksan | Erzurum Technical University | Harvard IRB approval sufficient | n/a |
| Uganda | Namutebi | Makerere University College of Humanities and Social Sciences | IRB approval granted | MAKSSREC 05.2023.680 |
| Ukraine | Ploszaj | University of Warsaw | Harvard IRB approval sufficient | n/a |
| United Kingdom | Bhatiya | University of Birmingham | Harvard IRB approval sufficient | n/a |
|  | Debnath | University of Cambridge | Declared exempt from full IRB review | 5051.145 |
|  | Douglas | University of Kent | IRB approval granted | 8016 |
|  | Alabrese | University of Bath | Harvard IRB approval sufficient | n/a |
|  | Ulug | University of Sussex | Harvard IRB approval sufficient | n/a |
| USA | Motta | Boston University | Declared exempt from full IRB review | H-43232 |
|  | Vilares | University of Minnesota | IRB approval granted | STUDY00005811 |
| Uruguay | Etienne | VU University Amsterdam | Declared exempt from full IRB review | 2023-1-30-546 |

**Supplementary Table 2. Overview of variables missing from single country samples**

| Construct | Item label prefix | Item label suffix | Measure | Not available for |
| --- | --- | --- | --- | --- |
| Willingness to be vulnerable to scientists | WILLVUL | ppl | How much or little should people rely on scientists’ guidance when making lifestyle choices related to science? | Finland, Mexico, Netherlands (917 out of 1,427 cases) |
|  |  | gov | How much or little should governments rely on scientists’ guidance when making decisions related to science? | Mexico, Netherlands (917 out of 1,427 cases) |
|  |  | you | How much or little control do you want scientists to have on government decisions related to science? | Mexico, Netherlands (917 out of 1,427 cases) |
|  |  |  |  |  |
| Normative perceptions of science and society | NORMPERC | communicate | To what extent do you disagree or agree that scientists should communicate their findings to politicians? | Argentina |
|  |  | independent | To what extent do you agree or disagree that scientists should remain independent from the policy-making process? | Malaysia |
|  |  |  |  |  |
| Exposure to information about science in news media | SCIINFO | newswebsitesapps | Over the past 12 months, how often have you come across information about science In news articles on news websites or in news apps? | Peru |
|  |  |  |  |  |
| Emotions about climate change | CLIM_EMO | anxious | To what extent does climate change make you feel anxious? | Albania |
|  |  | ashamed | To what extent does climate change make you feel ashamed? | Malaysia |
|  |  | indifferent | To what extent does climate change make you feel indifferent? | Malaysia |
|  |  |  |  |  |
| Support for environmental policies | CLIM_POLSUPPORT | fueltax | Please indicate your level of support for raising carbon taxes on gas and fossil fuels or coal. | Argentina, Malaysia |
|  |  | publictransport | Please indicate your level of support for expanding infrastructure for public transportation. | Argentina, Malaysia |
|  |  | sustenergy | Please indicate your level of support for increasing the use of sustainable energy such as wind and solar energy. | Argentina, Malaysia |
|  |  | protection | Please indicate your level of support for protecting forested and land areas. | Argentina, Malaysia |
|  |  | foodtax | Please indicate your level of support for increasing taxes on carbon intense foods (e.g., beef and dairy products). | Argentina, Malaysia |
|  |  |  |  |  |
| Perceptions of past extreme weather events | CLIM_WEATHERPAST | floods | To what extent do you think that climate change has increased the impact of floods over the last decades? | Albania |
|  |  | heatwaves | To what extent do you think that climate change has increased the impact of heatwaves over the last decades? | Albania |
|  |  | heavystorms | To what extent do you think that climate change has increased the impact of heavy storms over the last decades? | Albania |
|  |  | wildfires | To what extent do you think that climate change has increased the impact of wildfires over the last decades? | Albania |
|  |  | heavyrain | To what extent do you think that climate change has increased the impact of heavy rain over the last decades? | Albania |
|  |  | droughts | To what extent do you think that climate change has increased the impact of droughts over the last decades? | Albania |
|  |  |  |  |  |
| Perceptions of future extreme weather events | CLIM_WEATHERFUTU | floods | To what extent do you think that climate change will increase the impact of floods in the future? | Brazil, Mexico, Malaysia, Netherlands (917 out of 1,427 cases) |
|  |  | heatwaves | To what extent do you think that climate change will increase the impact of heatwaves in the future? | Brazil, Mexico, Malaysia, Netherlands (917 out of 1,427 cases) |
|  |  | heavystorms | To what extent do you think that climate change will increase the impact of heavy storms in the future? | Brazil, Mexico, Malaysia, Netherlands (917 out of 1,427 cases) |
|  |  | wildfires | To what extent do you think that climate change will increase the impact of wildfires in the future? | Brazil, Mexico, Malaysia, Netherlands (917 out of 1,427 cases) |
|  |  | heavyrain | To what extent do you think that climate change will increase the impact of heavy rain in the future? | Brazil, Mexico, Malaysia, Netherlands (917 out of 1,427 cases) |
|  |  | droughts | To what extent do you think that climate change will increase the impact of droughts in the future? | Brazil, Mexico, Malaysia, Netherlands (917 out of 1,427 cases) |
|  |  |  |  |  |
| Social dominance orientation | SDO | allgroupsconsider | How much do you favor or oppose the idea that in setting priorities, we must consider all groups? | Malaysia |
|  |  | notpushequality | How much do you favor or oppose the idea that we should not push for group equality? | Malaysia |
|  |  | equalityideal | How much do you favor or oppose the idea that group equality should be our ideal? | Malaysia |
|  |  | superiordominate | How much do you favor or oppose the idea that superior groups should dominate inferior groups? | Malaysia |

**Supplementary Table 3. Sample characteristics across countries, unweighted data (1)**

| Country | *n* |  | Gender | |  | Age | |  | Age group | | | | |
| --- | --- | --- | --- | --- | --- | --- | --- | --- | --- | --- | --- | --- | --- |
|  |  |  | %  female | %  male |  | *M* | *SD* |  | %  18-29 years | % 30-39 years | % 40-49 years | % 50-59 years | %  60+ years |
| Albania | 377 |  | 45.19 | 54.81 |  | 29.38 | 8.13 |  | 57.43 | 30.90 | 9.33 | 2.04 | 0.29 |
| Argentina | 509 |  | 52.53 | 47.47 |  | 43.18 | 14.35 |  | 20.00 | 22.63 | 21.62 | 20.40 | 15.35 |
| Australia | 3,560 |  | 50.21 | 49.79 |  | 45.51 | 16.38 |  | 19.98 | 20.01 | 20.04 | 19.93 | 20.04 |
| Austria | 1,076 |  | 51.11 | 48.89 |  | 44.92 | 15.07 |  | 19.61 | 19.32 | 19.81 | 21.45 | 19.81 |
| Bangladesh | 496 |  | 44.63 | 55.37 |  | 38.01 | 11.77 |  | 26.86 | 26.86 | 26.65 | 15.91 | 3.72 |
| Belgium | 2,052 |  | 51.20 | 48.80 |  | 45.69 | 15.51 |  | 17.59 | 21.72 | 20.39 | 20.39 | 19.9 |
| Bolivia | 548 |  | 47.46 | 52.54 |  | 38.29 | 11.76 |  | 26.17 | 26.37 | 26.17 | 19.47 | 1.83 |
| Botswana | 508 |  | 58.10 | 41.90 |  | 32.60 | 9.17 |  | 44.39 | 33.17 | 17.21 | 4.74 | 0.50 |
| Brazil | 1,336 |  | 51.57 | 48.43 |  | 40.51 | 13.64 |  | 25.04 | 25.45 | 22.65 | 16.23 | 10.63 |
| Bulgaria | 497 |  | 50.10 | 49.90 |  | 44.62 | 14.59 |  | 19.92 | 19.72 | 20.32 | 20.12 | 19.92 |
| Cameroon | 505 |  | 43.76 | 56.24 |  | 36.99 | 11.39 |  | 29.81 | 28.75 | 25.58 | 12.26 | 3.59 |
| Canada | 2,535 |  | 49.98 | 50.02 |  | 45.11 | 15.33 |  | 19.98 | 19.94 | 19.98 | 19.98 | 20.10 |
| Chile | 1,058 |  | 50.15 | 49.85 |  | 44.50 | 15.04 |  | 20.04 | 20.04 | 19.74 | 20.04 | 20.14 |
| China | 526 |  | 49.90 | 50.10 |  | 44.23 | 14.33 |  | 19.72 | 20.12 | 20.12 | 20.12 | 19.92 |
| Colombia | 514 |  | 49.90 | 50.10 |  | 44.86 | 14.84 |  | 20.04 | 19.64 | 20.04 | 20.04 | 20.24 |
| Congo DR | 408 |  | 49.61 | 50.39 |  | 39.84 | 13.29 |  | 28.79 | 24.16 | 18.77 | 17.22 | 11.05 |
| Costa Rica | 573 |  | 49.72 | 50.28 |  | 40.23 | 13.26 |  | 24.86 | 25.6 | 22.45 | 18.92 | 8.16 |
| Côte d’Ivoire | 509 |  | 40.56 | 59.44 |  | 43.81 | 14.41 |  | 28.11 | 29.18 | 26.82 | 13.73 | 2.15 |
| Cyprus | 502 |  | 48.80 | 51.20 |  | 44.97 | 15.23 |  | 21.12 | 19.52 | 20.72 | 20.52 | 18.13 |
| Czech Rep. | 514 |  | 50.30 | 49.70 |  | 37.29 | 11.27 |  | 19.60 | 19.80 | 20.20 | 20.20 | 20.20 |
| Denmark | 1,227 |  | 50.41 | 49.59 |  | 45.94 | 16.10 |  | 19.78 | 19.37 | 19.62 | 19.62 | 21.61 |
| Egypt | 512 |  | 48.81 | 51.19 |  | 40.37 | 12.10 |  | 23.81 | 23.81 | 23.81 | 23.10 | 5.48 |
| Ethiopia | 455 |  | 41.60 | 58.40 |  | 29.36 | 7.79 |  | 55.92 | 34.44 | 6.34 | 3.31 | 0.00 |
| Finland | 1,009 |  | 50.00 | 50.00 |  | 45.12 | 15.68 |  | 20.04 | 20.04 | 20.04 | 19.94 | 19.94 |
| France | 2,029 |  | 49.98 | 50.02 |  | 44.94 | 14.71 |  | 20.03 | 19.93 | 20.08 | 19.93 | 20.03 |
| Georgia | 528 |  | 49.90 | 50.10 |  | 44.51 | 14.48 |  | 19.47 | 20.08 | 20.28 | 20.08 | 20.08 |
| Germany | 8,134 |  | 50.02 | 49.98 |  | 45.06 | 15.27 |  | 19.99 | 19.99 | 19.98 | 19.99 | 20.05 |
| Ghana | 509 |  | 34.18 | 65.82 |  | 29.02 | 7.30 |  | 58.44 | 32.49 | 8.65 | 0.00 | 0.42 |
| Greece | 1,449 |  | 49.65 | 50.35 |  | 44.16 | 14.16 |  | 19.29 | 20.47 | 21.03 | 20.96 | 18.25 |
| Hong Kong | 599 |  | 49.83 | 50.17 |  | 44.47 | 14.60 |  | 19.70 | 20.20 | 20.20 | 20.37 | 19.53 |
| Hungary | 508 |  | 50.00 | 50.00 |  | 44.72 | 14.89 |  | 19.88 | 19.88 | 20.08 | 20.08 | 20.08 |
| India | 502 |  | 47.78 | 52.22 |  | 43.09 | 14.36 |  | 21.14 | 20.72 | 21.14 | 20.93 | 16.07 |
| Indonesia | 2,104 |  | 49.08 | 50.92 |  | 37.33 | 11.04 |  | 28.75 | 27.58 | 28.50 | 12.50 | 2.68 |
| Ireland | 506 |  | 50.00 | 50.00 |  | 44.43 | 15.15 |  | 19.88 | 19.88 | 20.08 | 20.08 | 20.08 |
| Israel | 1,049 |  | 49.80 | 50.20 |  | 44.75 | 15.32 |  | 20.10 | 20.00 | 19.90 | 20.10 | 19.90 |
| Italy | 1,520 |  | 50.10 | 49.90 |  | 45.17 | 15.12 |  | 20.20 | 19.87 | 20.00 | 20.00 | 19.93 |
| Japan | 1,004 |  | 50.00 | 50.00 |  | 44.71 | 15.33 |  | 20.00 | 20.00 | 20.00 | 20.00 | 20.00 |
| Kazakhstan | 520 |  | 44.05 | 55.95 |  | 40.88 | 13.43 |  | 23.59 | 23.59 | 23.98 | 17.93 | 10.92 |
| Kenya | 513 |  | 47.59 | 52.41 |  | 39.00 | 12.16 |  | 27.41 | 25.44 | 23.46 | 19.30 | 4.39 |
| Malaysia | 1,046 |  | 47.72 | 52.28 |  | 41.64 | 13.52 |  | 22.84 | 22.94 | 22.64 | 21.02 | 10.56 |
| Mexico | 532 |  | 49.40 | 50.60 |  | 44.13 | 14.40 |  | 20.28 | 19.48 | 20.48 | 19.68 | 20.08 |
| Morocco | 503 |  | 46.93 | 53.07 |  | 38.65 | 12.55 |  | 26.67 | 26.67 | 25.60 | 15.73 | 5.33 |
| Netherlands | 1,427 |  | 51.42 | 48.58 |  | 45.25 | 15.11 |  | 19.79 | 16.24 | 20.99 | 21.49 | 21.49 |
| New Zealand | 2,028 |  | 50.12 | 49.88 |  | 45.51 | 16.21 |  | 19.91 | 19.81 | 20.01 | 20.01 | 20.26 |
| Nicaragua | 499 |  | 46.12 | 53.88 |  | 39.25 | 12.34 |  | 24.81 | 24.81 | 25.06 | 20.80 | 4.51 |
| Nigeria | 1,040 |  | 50.30 | 49.70 |  | 34.56 | 11.92 |  | 41.60 | 27.23 | 16.60 | 12.25 | 2.33 |
| Norway | 513 |  | 50.61 | 49.39 |  | 46.22 | 16.09 |  | 19.03 | 20.24 | 20.24 | 20.24 | 20.24 |
| Peru | 513 |  | 50.00 | 50.00 |  | 44.38 | 14.90 |  | 19.96 | 19.76 | 20.16 | 20.16 | 19.96 |
| Philippines | 661 |  | 51.74 | 48.26 |  | 42.49 | 13.86 |  | 22.20 | 21.83 | 21.47 | 19.27 | 15.23 |
| Poland | 3,037 |  | 49.87 | 50.13 |  | 44.43 | 15.35 |  | 19.99 | 19.99 | 19.99 | 20.05 | 19.99 |
| Portugal | 502 |  | 50.10 | 49.90 |  | 44.68 | 14.54 |  | 19.84 | 20.04 | 19.84 | 20.04 | 20.24 |
| Romania | 444 |  | 35.88 | 64.12 |  | 44.41 | 14.71 |  | 18.75 | 23.38 | 20.83 | 19.21 | 17.82 |
| Russia | 1,518 |  | 50.10 | 49.90 |  | 44.24 | 14.51 |  | 19.96 | 20.09 | 20.03 | 19.96 | 19.96 |
| Serbia | 575 |  | 49.90 | 50.10 |  | 44.19 | 14.63 |  | 20.04 | 20.04 | 20.04 | 19.84 | 20.04 |
| Slovakia | 543 |  | 50.19 | 49.81 |  | 45.16 | 15.04 |  | 19.25 | 21.70 | 19.81 | 18.87 | 20.38 |
| Slovenia | 528 |  | 49.90 | 50.10 |  | 44.32 | 15.18 |  | 19.96 | 19.96 | 20.16 | 19.96 | 19.96 |
| South Africa | 1,027 |  | 50.00 | 50.00 |  | 44.49 | 15.04 |  | 20.00 | 20.00 | 20.00 | 20.00 | 20.00 |
| South Korea | 500 |  | 50.00 | 50.00 |  | 44.84 | 14.37 |  | 20.00 | 20.00 | 20.00 | 20.00 | 20.00 |
| Spain | 1,015 |  | 50.05 | 49.95 |  | 44.58 | 14.91 |  | 20.22 | 20.12 | 20.02 | 19.72 | 19.92 |
| Sweden | 1,013 |  | 49.90 | 50.10 |  | 45.98 | 16.11 |  | 19.92 | 19.92 | 20.02 | 20.22 | 19.92 |
| Switzerland | 1,018 |  | 49.95 | 50.05 |  | 44.72 | 14.91 |  | 19.96 | 20.06 | 19.96 | 20.06 | 19.96 |
| Taiwan | 1,206 |  | 50.00 | 50.00 |  | 44.19 | 14.47 |  | 20.27 | 20.02 | 20.02 | 19.93 | 19.77 |
| Türkiye | 508 |  | 50.00 | 50.00 |  | 44.26 | 14.21 |  | 20.00 | 20.00 | 20.00 | 20.00 | 20.00 |
| Uganda | 513 |  | 47.80 | 52.20 |  | 34.02 | 9.30 |  | 39.28 | 33.07 | 23.00 | 3.88 | 0.78 |
| Ukraine | 1,020 |  | 49.80 | 50.20 |  | 43.87 | 14.37 |  | 19.94 | 20.24 | 20.04 | 19.94 | 19.84 |
| UK | 2,008 |  | 50.03 | 49.97 |  | 45.20 | 15.27 |  | 19.67 | 20.12 | 20.07 | 20.07 | 20.07 |
| US | 2,580 |  | 54.20 | 45.80 |  | 47.41 | 16.86 |  | 17.04 | 20.13 | 17.98 | 16.84 | 28.02 |
| Uruguay | 325 |  | 55.77 | 44.23 |  | 52.41 | 12.83 |  | 4.81 | 13.78 | 19.23 | 29.81 | 32.37 |

**Supplementary Table 4. Sample characteristics across countries, unweighted data (2)**

| Country | Education | | | |  | Annual household income in USD | | | Political orientation (conservative) | | |
| --- | --- | --- | --- | --- | --- | --- | --- | --- | --- | --- | --- |
|  | % none | %  primary | % secondary | % tertiary |  | *Me* | *SD* |  | | *M* | *SD* |
| Albania | 0.87 | 1.46 | 14.58 | 83.09 |  | 2,319 | 510,141 |  | | 2.43 | 1.42 |
| Argentina | 0.00 | 2.02 | 38.59 | 59.39 |  | 1,523 | 236,584 |  | | 2.72 | 1.01 |
| Australia | 0.09 | 1.42 | 29.52 | 68.98 |  | 63,318 | 335,311 |  | | 3.41 | 1.16 |
| Austria | 0.00 | 3.29 | 62.90 | 33.82 |  | 26,779 | 61,079 |  | | 2.77 | 1.01 |
| Bangladesh | 0.00 | 1.03 | 15.08 | 83.88 |  | 1,932 | 7,268 |  | | 2.79 | 1.55 |
| Belgium | 0.00 | 2.06 | 37.74 | 60.20 |  | 52,855 | 103,878 |  | | 2.87 | 1.01 |
| Bolivia | 0.00 | 0.81 | 27.99 | 71.20 |  | 723 | 27,398 |  | | 3.14 | 1.43 |
| Botswana | 0.00 | 0.00 | 16.71 | 83.29 |  | 3,756 | 13,628 |  | | 3.43 | 1.35 |
| Brazil | 0.16 | 9.23 | 54.37 | 36.24 |  | 403 | 142,825 |  | | 3.32 | 1.40 |
| Bulgaria | 0.20 | 0.60 | 32.60 | 66.60 |  | 11,966 | 150,367 |  | | 3.09 | 0.91 |
| Cameroon | 0.00 | 0.42 | 18.39 | 81.18 |  | 9 | 68,954 |  | | 2.99 | 1.48 |
| Canada | 0.28 | 1.64 | 28.84 | 69.25 |  | 47,466 | 65,253 |  | | 2.94 | 1.19 |
| Chile | 0.20 | 1.20 | 28.41 | 70.19 |  | 2,504 | 3,829,874 |  | | 2.88 | 1.23 |
| China | 0.40 | 1.61 | 13.68 | 84.31 |  | 29,483 | 39,165 |  | | 2.71 | 1.01 |
| Colombia | 0.00 | 1.40 | 29.86 | 68.74 |  | 1,284 | 11,224 |  | | 2.74 | 1.14 |
| Congo DR | 0.00 | 0.00 | 8.23 | 91.77 |  | 3,600 | 101,931 |  | | 2.78 | 1.45 |
| Costa Rica | 0.00 | 4.45 | 36.36 | 59.18 |  | 2,493 | 170,383 |  | | 3.21 | 1.44 |
| Côte d’Ivoire | 0.21 | 0.21 | 16.09 | 83.48 |  | 16,079 | 160,967 |  | | 2.68 | 1.00 |
| Cyprus | 0.20 | 1.99 | 31.67 | 66.14 |  | 20,334 | 23,741 |  | | 3.21 | 0.93 |
| Czech Rep. | 0.20 | 3.23 | 63.23 | 33.33 |  | 1,613 | 14,356 |  | | 3.05 | 1.39 |
| Denmark | 0.17 | 18.38 | 26.08 | 55.38 |  | 50,989 | 118,321 |  | | 2.83 | 0.90 |
| Egypt | 0.95 | 0.24 | 14.29 | 84.52 |  | 2,618 | 4,494 |  | | 4.04 | 1.18 |
| Ethiopia | 0.28 | 0.28 | 16.80 | 82.64 |  | 1,115 | 913,162 |  | | 2.81 | 1.38 |
| Finland | 0.00 | 7.92 | 50.10 | 41.98 |  | 42,769 | 45,967 |  | | 2.85 | 1.09 |
| France | 0.10 | 0.55 | 34.03 | 65.32 |  | 26,641 | 40,661 |  | | 2.90 | 1.08 |
| Georgia | 0.20 | 2.43 | 32.66 | 64.71 |  | 18,869 | 117,218 |  | | 3.24 | 1.01 |
| Germany | 0.04 | 0.40 | 66.06 | 33.50 |  | 32,032 | 1,456,632 |  | | 2.92 | 0.99 |
| Ghana | 0.00 | 1.27 | 23.21 | 75.53 |  | 1,627 | 102,310 |  | | 3.12 | 1.40 |
| Greece | 0.21 | 2.99 | 51.88 | 44.92 |  | 15,988 | 75,894 |  | | 2.72 | 0.99 |
| Hong Kong | 0.00 | 2.36 | 30.98 | 66.67 |  | 62,170 | 65,735 |  | | 2.70 | 0.95 |
| Hungary | 0.00 | 5.62 | 66.67 | 27.71 |  | 8,412 | 21,146 |  | | 3.06 | 1.10 |
| India | 0.00 | 0.42 | 5.50 | 94.08 |  | 7,288 | 20,682 |  | | 3.18 | 1.31 |
| Indonesia | 0.10 | 0.10 | 22.57 | 77.24 |  | 4,355 | 26,329 |  | | 3.39 | 1.02 |
| Ireland | 0.00 | 2.41 | 36.95 | 60.64 |  | 48,423 | 308,541 |  | | 2.84 | 0.98 |
| Israel | 0.00 | 0.40 | 29.80 | 69.80 |  | 6,895 | 34,703 |  | | 2.52 | 1.08 |
| Italy | 0.00 | 0.33 | 61.93 | 37.74 |  | 29,427 | 59,649 |  | | 2.68 | 1.02 |
| Japan | 0.30 | 0.10 | 18.10 | 81.50 |  | 37,170 | 115,220 |  | | 3.14 | 1.03 |
| Kazakhstan | 0.19 | 0.00 | 40.74 | 59.06 |  | 3,227 | 416,321 |  | | 3.29 | 1.16 |
| Kenya | 0.22 | 0.66 | 15.13 | 83.99 |  | 2,616 | 21,373 |  | | 3.20 | 1.43 |
| Malaysia | 0.10 | 0.41 | 22.13 | 77.36 |  | 9,005 | 74,062,721 |  | | 3.00 | 0.78 |
| Mexico | 0.40 | 0.60 | 35.14 | 63.86 |  | 5,551 | 245,296 |  | | 2.77 | 1.25 |
| Morocco | 0.27 | 0.80 | 28.53 | 70.40 |  | 4,801 | 502,629 |  | | 3.70 | 1.26 |
| Netherlands | 0.07 | 2.55 | 51.35 | 46.03 |  | 45,804 | 173,423 |  | | 2.83 | 0.95 |
| New Zealand | 0.15 | 2.64 | 28.82 | 68.39 |  | 61,918 | 1,080,212 |  | | 3.43 | 1.15 |
| Nicaragua | 0.25 | 1.50 | 25.56 | 72.68 |  | 760 | 2,700 |  | | 2.96 | 1.35 |
| Nigeria | 0.00 | 0.30 | 13.36 | 86.34 |  | 2,609 | 242,274 |  | | 3.30 | 1.38 |
| Norway | 0.00 | 6.48 | 36.03 | 57.49 |  | 46,973 | 77,668 |  | | 2.95 | 1.06 |
| Peru | 0.00 | 0.00 | 24.40 | 75.60 |  | 2,663 | 40,164 |  | | 3.53 | 1.07 |
| Philippines | 0.18 | 0.18 | 18.35 | 81.28 |  | 4,279 | 16,594 |  | | 3.35 | 1.16 |
| Poland | 0.03 | 4.03 | 53.23 | 42.70 |  | 13,879 | 34,879 |  | | 2.87 | 1.22 |
| Portugal | 0.00 | 3.41 | 44.09 | 52.51 |  | 21,850 | 66,503 |  | | 2.72 | 0.79 |
| Romania | 0.00 | 0.23 | 14.35 | 85.42 |  | 9,950 | 33,341 |  | | 2.41 | 0.96 |
| Russia | 0.00 | 0.40 | 35.93 | 63.67 |  | 5,786 | 539,344 |  | | 3.20 | 1.04 |
| Serbia | 0.00 | 1.20 | 41.88 | 56.91 |  | 1,849 | 172,833 |  | | 2.71 | 1.13 |
| Slovakia | 0.00 | 3.77 | 49.62 | 46.60 |  | 15,908 | 15,311 |  | | 3.17 | 1.12 |
| Slovenia | 0.00 | 3.39 | 50.90 | 45.71 |  | 16,054 | 24,449,777 |  | | 2.63 | 1.14 |
| South Africa | 0.00 | 0.10 | 35.40 | 64.50 |  | 10,955 | 296,801 |  | | 3.23 | 1.20 |
| South Korea | 0.00 | 1.00 | 17.20 | 81.80 |  | 40,500 | 44,790 |  | | 3.08 | 0.87 |
| Spain | 0.20 | 5.15 | 61.94 | 32.71 |  | 22,822 | 147,093 |  | | 2.70 | 1.07 |
| Sweden | 0.10 | 7.77 | 43.33 | 48.80 |  | 46,437 | 62,088 |  | | 2.83 | 1.05 |
| Switzerland | 0.30 | 19.96 | 37.11 | 42.63 |  | 76,088 | 119,543 |  | | 2.83 | 1.09 |
| Taiwan | 0.00 | 1.41 | 15.45 | 83.14 |  | 32,515 | 52,489 |  | | 2.45 | 1.08 |
| Türkiye | 0.00 | 2.60 | 27.00 | 70.40 |  | 6,919 | 12,031 |  | | 2.97 | 1.26 |
| Uganda | 0.00 | 0.00 | 14.99 | 85.01 |  | 1,875 | 16,851 |  | | 3.19 | 1.45 |
| Ukraine | 0.10 | 0.69 | 22.92 | 76.29 |  | 3,265 | 7,018 |  | | 3.05 | 1.32 |
| UK | 0.05 | 0.55 | 33.43 | 65.96 |  | 42,930 | 157,299 |  | | 2.90 | 1.08 |
| US | 0.59 | 3.79 | 40.72 | 54.90 |  | 49,000 | 232,114 |  | | 3.22 | 1.35 |
| Uruguay | 0.00 | 0.96 | 37.50 | 61.54 |  | 2,306 | 95,739 |  | | 2.52 | 1.24 |

**Supplementary Table 5. Sample characteristics across countries, unweighted data (3)**

| Country | Political orientation (right) | |  | Religiosity | |  | Place of residence | |
| --- | --- | --- | --- | --- | --- | --- | --- | --- |
|  | *M* | *SD* |  | *M* | *SD* |  | %  rural | %  urban |
| Albania | 2.97 | 1.14 |  | 3.56 | 1.17 |  | 14.29 | 85.71 |
| Argentina | 3.35 | 1.10 |  | 2.75 | 1.33 |  | 9.49 | 90.51 |
| Australia | 3.44 | 1.08 |  | 2.82 | 1.41 |  | 23.30 | 76.70 |
| Austria | 2.97 | 0.95 |  | 2.28 | 1.26 |  | 50.63 | 49.37 |
| Bangladesh | 3.66 | 1.22 |  | 3.99 | 1.05 |  | 21.69 | 78.31 |
| Belgium | 3.14 | 1.09 |  | 2.08 | 1.19 |  | 53.86 | 46.14 |
| Bolivia | 3.51 | 1.19 |  | 3.49 | 1.17 |  | 15.21 | 84.79 |
| Botswana | 3.45 | 1.12 |  | 3.99 | 1.14 |  | 26.68 | 73.32 |
| Brazil | 3.35 | 1.39 |  | 3.64 | 1.23 |  | 12.11 | 87.89 |
| Bulgaria | 3.23 | 0.89 |  | 2.97 | 1.16 |  | 15.09 | 84.91 |
| Cameroon | 3.44 | 1.22 |  | 3.91 | 1.22 |  | 16.07 | 83.93 |
| Canada | 3.01 | 1.06 |  | 2.51 | 1.36 |  | 24.01 | 75.99 |
| Chile | 3.11 | 1.13 |  | 2.88 | 1.40 |  | 12.66 | 87.34 |
| China | 3.05 | 0.75 |  | 1.92 | 1.18 |  | 6.64 | 93.36 |
| Colombia | 3.16 | 1.18 |  | 3.34 | 1.36 |  | 9.42 | 90.58 |
| Congo DR | 3.24 | 1.49 |  | 4.16 | 1.18 |  | 3.34 | 96.66 |
| Costa Rica | 3.55 | 1.09 |  | 3.38 | 1.35 |  | 36.55 | 63.45 |
| Côte d’Ivoire | 3.12 | 0.84 |  | 3.18 | 1.26 |  | 11.37 | 88.63 |
| Cyprus | 3.38 | 0.97 |  | 1.94 | 1.26 |  | 9.16 | 90.84 |
| Czech Republic | 3.21 | 1.20 |  | 3.98 | 1.19 |  | 23.84 | 76.16 |
| Denmark | 3.01 | 1.08 |  | 2.18 | 1.18 |  | 28.64 | 71.36 |
| Egypt | 3.64 | 1.26 |  | 4.09 | 0.86 |  | 5.71 | 94.29 |
| Ethiopia | 3.37 | 1.13 |  | 4.04 | 1.01 |  | 14.33 | 85.67 |
| Finland | 3.13 | 1.08 |  | 2.15 | 1.19 |  | 24.35 | 75.65 |
| France | 3.12 | 1.17 |  | 2.02 | 1.19 |  | 47.04 | 52.96 |
| Georgia | 3.18 | 1.04 |  | 3.12 | 1.27 |  | 8.11 | 91.89 |
| Germany | 2.95 | 0.86 |  | 2.13 | 1.24 |  | 42.55 | 57.45 |
| Ghana | 3.39 | 1.17 |  | 4.15 | 1.06 |  | 22.78 | 77.22 |
| Greece | 3.06 | 0.83 |  | 3.09 | 1.27 |  | 13.65 | 86.35 |
| Hong Kong | 3.10 | 0.73 |  | 2.12 | 1.31 |  | 1.85 | 98.15 |
| Hungary | 3.16 | 1.11 |  | 2.28 | 1.24 |  | 30.32 | 69.68 |
| India | 3.70 | 1.05 |  | 3.88 | 1.10 |  | 14.16 | 85.84 |
| Indonesia | 3.50 | 0.84 |  | 3.82 | 0.82 |  | 15.66 | 84.34 |
| Ireland | 2.93 | 1.02 |  | 2.54 | 1.26 |  | 39.76 | 60.24 |
| Israel | 3.44 | 0.93 |  | 2.19 | 1.26 |  | 17.92 | 82.08 |
| Italy | 2.98 | 1.10 |  | 2.69 | 1.31 |  | 28.04 | 71.96 |
| Japan | 3.21 | 0.82 |  | 2.54 | 1.16 |  | 52.20 | 47.80 |
| Kazakhstan | 3.24 | 0.96 |  | 2.87 | 1.10 |  | 15.98 | 84.02 |
| Kenya | 3.40 | 1.14 |  | 3.96 | 1.08 |  | 16.89 | 83.11 |
| Malaysia | 3.08 | 0.67 |  | 3.81 | 1.06 |  | 20.71 | 79.29 |
| Mexico | 3.10 | 1.16 |  | 2.98 | 1.25 |  | 10.64 | 89.36 |
| Morocco | 3.41 | 1.04 |  | 3.64 | 0.98 |  | 9.07 | 90.93 |
| Netherlands | 3.14 | 1.08 |  | 1.97 | 1.27 |  | 44.33 | 55.67 |
| New Zealand | 3.43 | 1.08 |  | 2.89 | 1.43 |  | 20.76 | 79.24 |
| Nicaragua | 3.08 | 1.36 |  | 3.61 | 1.18 |  | 19.30 | 80.70 |
| Nigeria | 3.66 | 1.12 |  | 4.06 | 0.99 |  | 16.40 | 83.60 |
| Norway | 3.10 | 1.10 |  | 2.16 | 1.27 |  | 40.49 | 59.51 |
| Peru | 3.54 | 1.00 |  | 3.24 | 1.18 |  | 6.25 | 93.75 |
| Philippines | 3.71 | 0.96 |  | 3.70 | 1.03 |  | 34.13 | 65.87 |
| Poland | 3.12 | 1.18 |  | 2.78 | 1.27 |  | 22.85 | 77.15 |
| Portugal | 2.95 | 0.94 |  | 2.38 | 1.10 |  | 24.65 | 75.35 |
| Romania | 3.20 | 1.02 |  | 2.28 | 1.25 |  | 12.04 | 87.96 |
| Russia | 3.10 | 0.89 |  | 2.55 | 1.17 |  | 13.04 | 86.96 |
| Serbia | 2.86 | 0.99 |  | 3.13 | 1.28 |  | 18.04 | 81.96 |
| Slovakia | 3.06 | 1.02 |  | 2.97 | 1.34 |  | 33.77 | 66.23 |
| Slovenia | 2.89 | 1.05 |  | 2.50 | 1.37 |  | 34.53 | 65.47 |
| South Africa | 3.33 | 1.11 |  | 3.83 | 1.29 |  | 13.80 | 86.20 |
| South Korea | 3.17 | 0.92 |  | 2.27 | 1.34 |  | 8.20 | 91.80 |
| Spain | 2.81 | 1.11 |  | 2.26 | 1.24 |  | 21.01 | 78.99 |
| Sweden | 3.11 | 1.18 |  | 1.83 | 1.13 |  | 30.58 | 69.42 |
| Switzerland | 3.14 | 1.04 |  | 2.24 | 1.25 |  | 52.86 | 47.14 |
| Taiwan | 3.16 | 0.70 |  | 2.95 | 1.22 |  | 18.60 | 81.40 |
| Türkiye | 2.95 | 1.30 |  | 3.15 | 1.24 |  | 4.60 | 95.40 |
| Uganda | 3.40 | 1.23 |  | 4.17 | 0.96 |  | 22.48 | 77.52 |
| Ukraine | 3.28 | 1.14 |  | 2.83 | 1.19 |  | 16.57 | 83.43 |
| United Kingdom | 2.90 | 1.02 |  | 2.09 | 1.27 |  | 32.02 | 67.98 |
| United States | 3.44 | 1.26 |  | 3.28 | 1.43 |  | 35.91 | 64.09 |
| Uruguay | 2.70 | 1.24 |  | 2.24 | 1.37 |  | 8.33 | 91.67 |

**Supplementary Table 6. Share of respondents failing the attention checks across countries (percentage of country sample)**

| Country | % failed 1^st^ check  (write “213”) | % failed  2^nd^ check (select strongly disagree) |  | Country | % failed 1^st^ check  (write “213”) | % failed  2^nd^ check  (select strongly disagree) |
| --- | --- | --- | --- | --- | --- | --- |
| Albania | 3.6 | 35.6 |  | Israel | 3.1 | 9.9 |
| Argentina | 13.7 | 36.3 |  | Italy | 0.8 | 33.9 |
| Australia | 2.2 | 18.4 |  | Japan | 1.5 | 31.1 |
| Austria | 5.3 | 14.2 |  | Kazakhstan | 21.8 | 29.3 |
| Bangladesh | 2.7 | 38.7 |  | Kenya | 4.4 | 20.4 |
| Belgium | 5.8 | 18.6 |  | Malaysia | 1.7 | 42.8 |
| Bolivia | 10.5 | 35.1 |  | Mexico | 8.6 | 36.3 |
| Botswana | 3.7 | 16.7 |  | Morocco | 2.3 | 32.7 |
| Brazil | 10.4 | 38.1 |  | Netherlands | 1.7 | 25.9 |
| Bulgaria | 6.9 | 27.6 |  | New Zealand | 2.0 | 19.2 |
| Cameroon | 4.6 | 17.7 |  | Nicaragua | 10.3 | 27.6 |
| Canada | 2.4 | 19.5 |  | Nigeria | 2.1 | 16.6 |
| Chile | 8.3 | 28.1 |  | Norway | 3.0 | 22.6 |
| China | 2.2 | 24.4 |  | Peru | 4.6 | 27.8 |
| Colombia | 4.9 | 24.1 |  | Philippines | 2.5 | 39.2 |
| Congo DR | 1.6 | 14.3 |  | Poland | 1.6 | 33.4 |
| Costa Rica | 10.8 | 31.6 |  | Portugal | 0.0 | 23.8 |
| Cyprus | 5.4 | 29.2 |  | Romania | 0.7 | 4.1 |
| Czech Republic | 1.8 | 34.7 |  | Russia | 16.1 | 11.7 |
| Côte d’Ivoire | 3.3 | 21.3 |  | Serbia | 2.2 | 19.7 |
| Denmark | 3.5 | 26.3 |  | Slovakia | 0.6 | 15.2 |
| Egypt | 2.7 | 14.3 |  | Slovenia | 4.0 | 30.6 |
| Ethiopia | 8.5 | 35.9 |  | South Africa | 5.7 | 19.6 |
| Finland | 2.2 | 17.7 |  | South Korea | 0.5 | 30.9 |
| France | 2.3 | 19.3 |  | Spain | 3.9 | 28.2 |
| Georgia | 26.3 | 38.2 |  | Sweden | 2.8 | 23.4 |
| Germany | 3.5 | 12.0 |  | Switzerland | 3.6 | 23.3 |
| Ghana | 1.9 | 26.3 |  | Taiwan | 1.2 | 20.4 |
| Greece | 1.5 | 31.0 |  | Türkiye | 2.0 | 44.1 |
| Hong Kong | 1.2 | 30.1 |  | Uganda | 3.4 | 27.3 |
| Hungary | 1.9 | 34.5 |  | Ukraine | 3.2 | 26.7 |
| India | 3.6 | 43.1 |  | United Kingdom | 1.5 | 10.9 |
| Indonesia | 0.7 | 23.5 |  | United States | 4.1 | 26.9 |
| Ireland | 2.3 | 31.0 |  | Uruguay | 1.0 | 7.2 |
| *Note*: Respondents in Georgia were filtered by the first attention check if they provided the correct answer in Georgian numerals. Similarly, respondents in Kazakhstan and Russia were filtered if they provided the answer in Cyrillic numerals. | | | | | | |

**Supplementary Table 7. Polychoric Exploratory Factor Analysis with the 12-item scale measuring perceived trustworthiness of scientists**

|  | Factor 1 | Factor 2 | Factor 3 | Factor 4 | Factor 5 |
| --- | --- | --- | --- | --- | --- |
| *Competence* |  |  |  |  |  |
| Expert |  | 0.60 |  |  | -0.26 |
| Intelligent |  | 0.77 |  |  |  |
| Qualified to conduct high-quality research |  | 0.75 |  |  |  |
| *Integrity* |  |  |  |  |  |
| Honest | 0.74 |  |  |  |  |
| Ethical | 0.44 |  |  |  |  |
| Sincere | 0.65 |  |  |  |  |
| *Benevolence* |  |  |  |  |  |
| Concerned about people’s wellbeing |  |  |  | 0.66 |  |
| Eager to improve others’ lives |  |  |  | 0.73 |  |
| Considerate of others’ interests |  |  | 0.49 |  |  |
| *Openness* |  |  |  |  |  |
| Open to feedback |  |  | 0.43 | 0.27 |  |
| Willing to be transparent | 0.34 |  | 0.33 |  |  |
| Pay attention to others’ views |  |  | 0.89 |  |  |
| *Note*: EFA used oblique rotation (oblimin) and principal axis factoring. Loadings < \|.25\| not displayed. | | | | | |

**Supplementary Table 8. Polychoric Exploratory Factor Analysis with the SciPop Scale measuring science-related populist attitudes**

|  | Factor 1 | Factor 2 | Factor 3 | Factor 4 |
| --- | --- | --- | --- | --- |
| *Conceptions of the ordinary people* |  |  |  |  |
| Ordinary people have in common that they trust their common sense in everyday life. |  |  |  | 0.73 |
| Ordinary people are of good and honest character. |  |  |  | 0.44 |
| *Conceptions of the academic elite* |  |  |  |  |
| Scientists are only interested in their own advantage. | 0.29 |  | 0.53 |  |
| Scientists are in cahoots with politicians and businesses. |  |  | 0.82 |  |
| *Demands for decision-making sovereignty* |  |  |  |  |
| Ordinary people should have influence on the work of scientists. |  | 0.81 |  |  |
| Ordinary people should be involved in decisions about the topics scientists research. |  | 0.73 |  |  |
| *Demands for truth-speaking sovereignty* |  |  |  |  |
| Ordinary people should trust their life experience more than the recommendations of scientists. | 0.72 |  |  |  |
| Our society should rely more on common sense than on scientific studies. | 0.76 |  |  |  |
| *Note*: EFA used oblique rotation (oblimin) and principal axis factoring. Loadings < \|.25\| not displayed. | | | | |
